# AI driven multi modal deep learning system for wheat disease detection, yield prediction, and crop health monitoring

**DOI:** 10.3389/fpls.2026.1769915

**Published:** 2026-08-13

**Authors:** Priyanshu Priyadarshi, Ganesh Khekare

**Affiliations:** School of Computer Science and Engineering, Vellore Institute of Technology, Vellore, Tamil Nadu, India

**Keywords:** agroinformatics, ConvNeXt, EfficientNet-B0 + CBAM, EfficientNet, mixed precision, wheat analytics

## Abstract

Sustainable wheat farming is challenging. Real-time information on crop health, disease transmission, and anticipated yields is essential for farmers. However, they frequently use slow, expensive, or non-communicative tools. This project develops a workable solution. There is no need for massive server farms because the entire system operates on a single graphics card. It incorporates images of wheat fields, Indian farming notes, greenhouse records, harvest statistics, and NASA meteorological data. Consider them as various “eyes” for crop photo analysis, and we tried several lightweight computer vision models. ConvNeXt-Tiny was slower but could operate on older equipment with 75% accuracy; EfficientNetB0 recognised wheat heads with 92% accuracy; and AgroMark, a hybrid solution that merged photo analysis with agricultural metadata (soil type, rainfall, increased to 87%, etc. Combining picture analysis with attention mechanisms (CBAM) allowed us to anticipate the amount of wheat that a field will yield based on these photo insights, and the results showed that our predictions were accurate, with an R^2^ score of 0.97. Additionally, we developed a versatile detector that simultaneously detects disease, stress, head count, and pests. It is adjusted to deal with training data that is unbalanced (some diseases are common, while others are rare). As we packed everything into a 16-GB graphics card, we spent real time determining which strategies smaller training sets, removing weak features, and adjusting loss functions, work. We encounter real-world obstacles along the road, such as photographs from different locations not always match, mislabeled photographs from different locations not always match, mislabeled diseases, and neglected rare pests. Our step-by-step instructions, charts, and code are available.

## 1 Introduction

Wheat secures food security for more than one-third of the global population and remains a strategic crop for India. In recent seasons, volatile rainfall, heat stress events and biological threats have pushed several regional yields below trend projections. Production shortfalls ripple quickly into price instability and threaten household income in agrarian economies. These methods are labour-intensive, cover limited spatial footprints and lag the pace at which pests and abiotic stress propagate. Recent advances in computer vision and machine learning offer a path to fuse imagery, climate logs and management records to deliver timely interventions; however, reproducible pipelines that run within the computational limits of shared research infrastructure are still scarce.

First, none of the examined studies combines vision, tabular learning, and reinforcement learning into a single versioned environment that can run on a commodity 16 GB GPU, and most of them don’t share any reusable code. Second, there is a significant class imbalance in disease datasets; minority pathogens like Tan spot (241 images) and stem fly (234 images) make up less than 2% of training data, yet no previous study reports per-class recall or eliminates focal loss, class weights, and augmentation techniques. Third, without using VIF filtering or grouped cross-validation, tabular yield models often incorporate target-correlated proxy features before train-test separation, unnecessarily inflating R² by 20–40 percentage points. Fourth, prediction is treated as the terminus in all reviewed research. Output, leaving intervention time up to human judgment and requiring little work to relate perceptual outputs to a reinforcement learning agent. These drawbacks underscore the necessity of a cohesive, resource-efficient, and reproducible multimodal AI system for wheat crop monitoring. In this study, a unified analytics stack is developed and implemented end-to-end on the Paper space Gradient platform. Three complementary modalities are used in the study: (i) canopy imagery for foliar disease identification and head counts; (ii) tabular descriptors from FAOSTAT production and Indian management statistics, NASA POWER climatology, Eschikon greenhouse sensors, and a reinforcement learning simulator that shows how perception modules can influence pesticide scheduling. Every module has been designed with repeatability in mind; every caching operation, dependency programmed to make it easy for peers to extend or replay the analysis.

### 1.1 AI-based techniques vs. conventional agronomic methods

Conventional wheat disease diagnosis relies on skilled plant pathologists undertaking field scouting, usually two to four surveys per growing season at the approved Zadoks growth stages (GS31, GS39, GS55, GS75). A skilled scout can visually evaluate five to ten fields every day for about ₹800–1,200 per field survey in India, or an estimated ₹2,000–3,000 per hectare for season-long monitoring. Standard rating criteria (e.g., modified Cobb scale for rust, 0–100% leaf area damaged) are used to measure disease severity; nevertheless, they are subject to observer fatigue and inter-rater variability of ±15–20% in field conditions and require years of training to apply consistently (Mapuranga and Yang, 2026).

In India, traditional yield forecasting is based on Crop Cutting Experiments (CCEs), a government-mandated technique where the ground-truth yield estimate used for crop insurance and procurement planning is obtained by harvesting a specific area, usually 5 m × 5 m. According to the World Bank, CCEs are labour-intensive (requiring 3–4 person-days per estimate), statistically noisy (coefficient of variance 20–35% at the district level), and only available after harvest, giving supply chain planners no early notice. AI-based methods have several complementary benefits:

- EfficientNetB zero (Tan and Le, 2019) detects wheat heads with 92% accuracy in less than 200 ms per image, compared to several hours per field for manual scouting.
- Yield prediction models trained on NASA POWER climate data can produce district-level forecasts 6–8 weeks before harvest at nearly zero marginal cost per prediction, compared to ₹1,500–2,000 per CCE; and
- AI models offer consistent, operator-independent evaluations that eliminate inter-rater variability. But AI methods are not a comprehensive replacement: they lack the agronomist’s capacity to evaluate soil structure, root health, and the socioeconomic background of specific farm management choices. The most promising approach is human-AI cooperation, in which agronomists concentrate specialist attention on identified high-risk fields while AI manages high-frequency, wide-area surveillance.

Important Machine Learning Ideas Employed in This Research (with Agricultural Context):

Convolutional Neural Network (CNN): This deep learning architecture learns spatial feature detectors, or filters, that are applied throughout the image. Agricultural application: uses field photos to identify signs of pest damage, wheat head morphologies, and disease lesion patterns (Goluguri and Baik, 2026).

Transfer learning is the process of starting a model with weights that have been pre-trained on a sizable, broad dataset (ImageNet) and then tailoring it to a particular domain. Agricultural application: by inheriting general visual feature detectors, disease detection models may be constructed using just about 13,000 wheat photos instead of millions. by inheriting general visual feature detectors, as opposed to millions.

Focal Loss: A variation on regular cross-entropy loss that forces the model to concentrate on uncommon/difficult cases by down-weighting simple samples during training.

Agricultural application: stops a disease classifier from favouring Brown Rust (1,301 photos) over Stem Fly (234 training images).

By interpolating between actual cases, SMOTE (Synthetic Minority Over-sampling Technique) creates artificial training examples for minority classes.

When low-intensity use data are underrepresented in district statistics, agricultural application balances the pesticide risk categorization training set.

Multicollinearity between predictor variables is measured by VIF (Variance Inflation Factor); features with VIF > 10 are eliminated. Agricultural application: eliminates strongly linked climate variables that would otherwise result in inflated model coefficients, such as maximum temperature and dew-point temperature.

Q-Learning: Reinforcement Learning): A model-free method that uses trial and error to teach an agent how to maximize cumulative reward. Agricultural application: based on current disease risk probability and climate anomalies, determines when to suggest pesticide action versus waiting.

The Attention Mechanism (CBAM) enables a neural network to dynamically determine which feature channels (channel attention) and which areas of an image (spatial attention) are more informative for a particular prediction task. Agricultural application: concentrates the yield prediction model on areas of the canopy that are impacted by disease instead of the background.

Gradient-weighted Class Activation Mapping, or Grad-CAM, shows which areas of an image were most important to a model’s prediction. Agricultural application: allows agronomists to confirm the biological plausibility of model decisions by displaying which leaf regions or head characteristics the disease classifier focuses on.

### 1.2 Problem statement

Despite the availability of large open datasets, deploying multi-modal wheat analytics in practice is hindered by three barriers. First, vision datasets are heavily imbalanced, with certain diseases registering fewer than 250 training images. Models trained naively tend to collapse, resulting in near-random predictions. Second, agronomic tables mix strongly correlated variables and target leakage (for example, the inclusion of yield while attempting to predict yield). Third, agricultural researchers frequently rely on notebook environments with limited GPU memory (Puglisi et al., 2026). Without deliberate sub-sampling, mixed precision and caching strategies, training modern networks such as Conv Next or Efficient Net becomes unstable (Zang et al., 2026). These limitations need to be overcome to make the system capable of making superior decisions.

The main objectives of this research work:

1. To create a standard and reproducible data integration pipeline that combines heterogeneous agricultural datasets, such as images, meteorological, and tabular data, ensuring consistency, annotated correctness, and preprocessing efficiency under limited computational resources.
2. To assess the effectiveness of lightweight deep learning architectures in wheat head detection and disease classification by measuring the performance of EfficientNet, ConvNeXt-Tiny, and attention-based architectures in terms of accuracy, weighted F1-measure, and robustness to class imbalance problems.
3. To create a hybrid prediction system that combines visual features extracted from crop images with structured agricultural features to achieve accurate wheat yield estimation, as well as measuring the performance using various regression metrics and feature interpretability techniques.
4. To develop a decision model using the reinforcement learning technique that can utilize perception-based inputs to optimise the strategy of using pesticides as interventions, as well as their convergence characteristics.
5. To develop an integrated and efficient system for agricultural analytics that can conduct simultaneous operations such as crop detection, disease identification, stress analysis, and yield prediction with reproducibility and within the context of a single-GPU environment.

The research work focuses on open datasets accessible without commercial restrictions.

The main materials are:

- The challenge of GWHD is now available. Head detection images with annotation will be used to establish the viewing conditions.
- All twenty-one brain diseases. Fifteen example settings illustrate the class imbalance problems addressed in the notebook.
- NASA POWER meteorology records and Eschikon greenhouse stress records. These two sources, the indices of which provided the climatic and physiological descriptors for the tabular models.
- FAOSTAT production statistics, and Indian agricultural records. National and district indicators provide the management context for yield and pesticide analyses.

This implementation for the Gradient cloud (one NVIDIA A4000 GPU, 16 GB RAM) relies on Python libraries such as TensorFlow 2.15, scikit-learn 1.7, etc. Model creation, ablations, and evaluations are required to reproduce the resource envelope available to Gradient and other participants, limited to this environment.

The following is a summary of this study’s major contributions:

- Fully scripted Paperspace Gradient notebook which accelerates the ramp up time by over 60% by scripting the dependency bootstrap, dataset checking and caching.
- The GWHD subset (1,303 pictures for test) of pictures were tested with the lightweight computer vision models (EfficientNetB zero, ConvNeXt Tiny, AgroMark).
- EfficientNetB zero (Tan and Le, 2019) achieves 92.0% ± 2.1% accuracy (95% CI: 89.2–94.8%), ConvNeXt Tiny achieves 88.8% ± 3.0%, and AgroMark achieves 91.2% ± 2.5%, across N = 3 independent runs under Gradient resource constraints. Within our experimental design these findings are competitive, however as we used several training methods, why us is limited when comparing to any published GWHD standards. See **Table 1** for complete metrics with confidence intervals and p-values.
- Leakage-free engineering of tabular predictors for yield and pesticide outcomes, including ensemble baselines Random Forest, XG-Boost, and Kolmogorov–Arnold networks.
- Integration of a Q-learning agent (Watkins and Dayan, 1992) that receives perception- derived signals and demonstrates reward convergence near 1.85 × 103, showcasing how analytics modules can inform agronomic policy.
- An extensive ablation and sensitivity study quantifying the effects of dataset sub-sampling, feature removal and reward scaling, accompanied by reproducible figures and tables.

**Table 1:** The combined test metrics for vision models with ROC AUC are included in binary rows, whereas weighted F1 and top-3 accuracy are reported in multi-class rows (David et al., 2020).

Task	Model	Accuracy	Precision	Recall	F1	AUC/Top- 3
GWHD classification	EfficientNet- B0	0.92	0.92	0.92	0.92	0.94
GWHD classification	ConvNeXt Tiny	0.888	0.89	0.89	0.89	n/a
GWHD classification	AgroMark fusion	0.912	0.91	0.91	0.91	n/a
Disease classification	EfficientNet- B3	0.9107	0.89	0.91	0.8923	0.9787
Disease (multi-task)	EfficientB0+C	BAM0.93	0.92	0.91	0.92	n/a
Multi-task detection	FastCNN	0.85–0.92	–	–	0.85–0.92	n/a

In Part 2 we analyze the state-of-the-art crop management tools based on image-models, table-databases, and trial-and-error. Chapter 4 describes where the data comes from and how it was classified, cleaned, and prepared, as well as how test station condition was monitored for consistency. Chapter 5 details how the model was constructed decisions on its architecture, its methodology, and hurdles encountered in real-world deployment. Chapter 6 considers the results all tests are described individually, camera images and data are interpreted. Finally, in Chapter 7, we consider conclusions what was learned and what the obstacles to success were. In the post-sections, additional content like measurement explanations, sample placement, and categorized data subsets are contained. To satisfy strict academic requirements, it is also a guide for researchers and students to evolve from raw data to deployable instrument.

## 2 Literature review

### 2.1 Vision-based wheat analytics

First attempts at wheat head detection using only monocular features had limited success. The failure to generalize to different wheat varieties or outdoor lighting conditions meant they fell back on handcrafted texture features and antiquated classifiers. The arrival of the Global Wheat Head Detection data (20120 (David et al., 2020)) ushered in a wave of deep convolutional models. Building blocks like EfficientNet (Tan and Le, 2019) offered a successful starting point by balancing network depth, width, and input resolution simultaneously under a compute budget. The technique was quickly adopted as a baseline by many research groups. More recent work saw the focus shift to a scaled down version of ConvNeXt (Liu et al., 2022) with large filters, depth wise splits, and residual skips inspired by transformer architectures. Training with ImageNet weights accelerated the network convergence.

Image classification was joined by augmentations involving flips, rotations, and hue variations, something that had repeatedly been shown to be effective. the previous five years. A 15-class reference corpus from Indian farms was assembled by the researcher (Nazir, 2020). To solve class imbalance, a number of Kaggle solutions stacked Efficient Net with focal loss; nonetheless, there is still a dearth of peer-reviewed research on reproducibility. Hybrid techniques that combine convolutional backbones with attention modules have also been tested by the agricultural computer vision community; however, these designs usually require more memory than field-deployable systems can supply. In order to stay inside the 16 GB GPU limit, our research focuses on light backbones and repeatable subsampling. Gathering clinical data on wheat blights has only been possible in our era and this could be one of the reasons why there are few studies into their classification.

Real farms outperform lab models if local seasons and climates are ignored in training data. Even though the top hits claim that more than 90% of crops are healthy. To increase test scores, some researchers fabricate data. When soils shift or climates change, impressive results from covert laboratories break apart. High-speed internet and durable gear for advanced instruments are lacking on remote fields. DIY picture tags increase expenses and facilitate mistakes. Forecasts from a proprietary technology are mistrusted by farmers.Local prediction algorithms are confused by mismatched surroundings, regional climates, soils, and pests. Improved infrastructure, reasonably priced equipment, training, and designs that take into account the reality of farming are all necessary for success; technological fixes alone won’t cut it (Aviles Toledo et al., 2024).

Using 400 wheat plots of five different types treated with various fertilisers, researchers assessed 25 regression models with many more samples than they typically use. In May and June, Support Vector Regression achieved R^2^ values of 0.95 and 0.91, respectively, while neural networks achieved 0.94, demonstrating that both conventional and deep learning are effective when properly calibrated. By automating the process, tools such as PyCaret avoided the need for human expertise. Basic greenness was exceeded by rededge and chlorophyll indices, with early May data (R^2^ = 0.95) showing the best results for prompt decision-making. However, the one-site, one-season approach restricts its wider European application (Berlingeri et al., 2025).

Transformers achieved a claimed accuracy of 99.8% in a study of 194 papers that looked at deep learning for wheat hyperspectral analysis; nevertheless, the results are suspiciously high and raise concerns about overfitting. Although hyperspectral imaging with more than 100 spectral bands can identify stress and illness before RGB photography, the equipment is more expensive than $10,000, and data preparation is difficult. Folks mostly used supervised approaches like CNNs, RNNs, and Transformers - alongside semi-supervised ones such as pseudo-labelling or GANs. For real-world use, clear public data sets matter; so does low-cost gear, plus model’s regular farmers can make sense of even if they’re less precise (Roy et al., 2025).

Just by adjusting the distance between plants, one harvest out of every seven grew larger. Examining 1,500 studies from Chinese farms revealed that crop spacing was more important than anticipated. Not the soil work, which was helpful, but properly managed roots also had a significant impact. Every technique produced improvements: improved ground underfoot increased productivity by 15%. Properly cared-for roots added 7. Changes in spacing helped 9. Two real-world winners include precise nutrient management for irrigated areas, which achieved 57% nitrogen efficiency, and plastic mulching for drylands. However, 34 kg of plastic waste each hectare necessitates cleanup or biodegradable alternatives. Local soils and meteorological conditions require that practices be modified. Adoption is influenced by communities, markets, incentives, and regulations in addition to technology. Though they require extensive testing, emerging AI technologies like XGBoost and graph networks promise more precise forecasts (Borisov et al., 2024).

Researchers looked at more than 140 research on machine learning for Indian farmers who use satellite imagery to inform crop choices. They discovered that support vector machines can forecast yields with little data, while random forests are excellent at recognising crops (85–95%). Neural networks gained popularity after 2018 thanks to improved datasets and affordable cloud computing, but most of them ignore the suffering farmers in arid central India in favour of the bountiful Indo-Gangetic Plains. The most common imaging is Landsat/Sentinel, with hyperspectral imaging developing for nutrient and pest identification (Xu and Wang, 2025). The true problem is that models are devoid of adoption studies, economic analysis, and field validation. Affordable, farmer-involved systems must be given priority over algorithms in future research.

68% of the 262 machine learning studies for farming that researchers examined between 2018 and 2020 focused on crop management issues that farmers care about, such as yield forecasting, disease detection, and pest control. Since artificial neural networks can handle chaotic farm data without requiring complex expert preparation, they seized the lead. As computers became more affordable, the field expanded by 745%. To create smarter models, teams used satellites, drones, and ground sensors; nevertheless, coordination issues and data format conflicts caused delays. While random forests performed well for weather-soil-management problems where knowing the “why” and having limited data were important, deep learning excelled in disease diagnosis. The main issue is that most research focuses on large, wealthy farms while neglecting tiny, developing-world businesses, and lab triumphs fail in real-world settings (Kaur et al., 2025).

When using red and near-infrared satellite data, which better reveal plant stress than basic color imaging, researchers found that LSTM networks achieve an R^2^ of 0.93, significantly outperforming gradient boosting (0.86) and support vector regression (0.57). This was based on an analysis of 119 studies on deep learning for wheat yields. For prompt management decisions, midseason forecasting is ideal. However, models developed in developed nations frequently don’t function well in developing regions because of environmental variances, and because they are opaque, farmers are reluctant to trust and utilize them (Kesavan et al., 2025).

Researchers looked at 262 papers that demonstrated how cameras and machine learning could detect plant stress far more quickly than human vision. Using inexpensive RGB cameras that cost about $100, convolutional neural networks were able to detect diseases with above 90% accuracy in 135 trials. Support vector machines handled small datasets well in 84 studies. Random forests explained which plant features mattered most. Hybrid methods combine strengths for better results. Hyperspectral sensors caught subtle issues, but cost over $10,000. Readings were distorted by lighting and shadows, and widely used databases such as Plant Village ignored rare diseases in favour of overemphasizing common ones. One season at a single location, red and near-infrared sensors delivered stronger results than standard greenness indicators. Testing six machine learning methods on drone images from German wheat fields in 2020 revealed that random forest along with gradient boosting outperformed simpler approaches. Basic decision trees lagged behind - reaching only an R^2^ of 0.63. Support vector regression did somewhat better at 0.72. Performance jumped when model settings were fine-tuned, lifting accuracy between 7 and 12 percent. Still, how well these findings apply beyond the test area remains uncertain. Year-round conditions such as rain levels, heat shifts, and fertilizer use play major roles in crop output. Because those elements vary widely, checking results across different regions and growing periods becomes necessary. Promising outcomes in one setting do not guarantee repeat success elsewhere (Liu et al., 2022).

In their Final paper, researchers tested whether LSTM neural networks can make use of satellite vegetation data from 15,709 wheat fields in China and achieved greatly exceeding random forest, gradient boosting, or support vector regression (R^2^ = 0.93). These predictive models learned temporal crop development trajectories and captured the shoot early stress result on final yields. SWIR signals excelled over simply greenness for prediction. While 30-meter resolution satellite imagery is inferior to 10-meter imagery, the variation in yields induced by management practice such as seed rates, nitrogen timing, and irrigation was only 15-25%. 30% of the data was lost due to cloud cover and the. ground truth collection from only 39 sites may only have a minimal coverage of Chinese farms (Liu et al., 2024).

Australian farmers deployed drones and machine learning daily, to monitor wheat fields, instead of waiting for weekly satellite flybys, which was earlier of great help in early detection of pest outbreaks and nutrient issues. Random forest algorithms could detect a variety of crop stressors in 87% of testing cases. Variable rate fertilizer application has reduced their spending by 15 22%, while keeping yields steady. Their water use was cut by 12 18%, in smart irrigation. However, there were significant barriers to uptake, with the costs of flight equipment, the services of a competent operator, software licenses, and weather setbacks, when disease pressure was high. For smallholder farms, the critical factors were expenditure and the complexity of operation (Kumar et al., 2025).

To predict wheat yields, researchers combined weather, genetics, soil, and farm management techniques using random forest models. This resulted in an R^2^ of 0.76 84, which was much higher than just soil or weather alone. Working together with farmers improved the model and gained their trust. Adoption was hampered by a shortage of funds, unstable land tenure, and loan availability more than ignorance. Despite overseeing several hectares, women farmers received little assistance from the extension department. Forecasts that are flexible rather than prescriptive are necessary due to climate variability. Extension personnel must be trained in contemporary sustainable farming techniques in order to scale (Ministry of Agriculture and Farmers Welfare, Government of India, 2020).

Researchers looked at more than six vegetation indices from multispectral and hyperspectral imagery to identify wheat disease, nutritional problems, and water stress; red-odge and chlorophyll indices outperformed standard greenness measures; shortwave infrared detected water stress with 82% accuracy versus 65% for basic indices; nitrogen deficiency showed up to three to five days early in spectral signatures before visible symptoms; disease types had distinct spectral fingerprints but overlapped; multispectral imagery offered a practical balance between sensing capability and cost; however, and geographical variations required site-specific calibration (Nazir, 2020).

Case studies from 47 farms show that machine learning decision support systems had real acceptance problems despite their technological capabilities. Farmers needed training and expertise with software. Extension agents were unable to offer continuous assistance. 5–12% of smallholder income was consumed by technology expenses. Farmers were concerned about losing their information advantage if they shared field data. Cloud systems were blocked by inconsistent electricity and poor internet. The black-box projections made by deep learning eroded confidence. Successful implementations included human experience, straightforward interfaces, compatibility with existing processes, and a staggered deployment that allowed farmers to learn and adapt (Priyadarshi et al., 2025).

Researchers looked into transfer learning techniques to modify disease detection algorithms for new agricultural scenarios with little local data. ImageNet was outperformed by 20–30% by pretraining on agricultural datasets. Style transfer and generative networks improved accuracy by 12–15% by bridging the gaps between lab and field photos. Few-shot learning identified illnesses with 75–82% accuracy from just 5–10 examples. On unlabeled field photos, self-supervised learning improved performance by 8–12%. Farmers’ labeling labor was reduced by 30–40% thanks to active learning. Meta-learning quickly adjusted to novel illnesses. However, navigating still-different farm conditions remained challenging (Salka et al., 2025).

Researchers experimented with a number of augmentation strategies to boost training data for illness detection when field samples were expensive and hard to come by. Images that were cropped and rotated were somewhat helpful. Changes in color brightness increased robustness by 4-7%. Generative network-generated synthetic images increased accuracy by 8–12%. The most accurate illness simulations were those based on physics, which increased accuracy by 15–20% by displaying the course of the disease over time. Although copy-paste lesions appeared realistic, they occasionally backfired. Rare diseases benefited by class balancing. Ten to eighteen percent of camera differences were adjusted for via style transfer. However, excessive distortion had a detrimental effect on performance, and efficacy varied according on the illness and situation (Shafay et al., 2025).

Farmers wanted to know why models generated forecasts rather than just accepting black-box facts. The crop qualities that were most important were indicated by the feature importance rating. Disease detection was explained by highlighting important areas of the image. Farmers were able to identify judgments with the aid of similar training instances. What-if research revealed small adjustments that reversed forecasts. There is no doubt that decision trees explained themselves. Farmers can investigate how inputs impacted outputs with interactive tools. It was verified that models accurately depicted biological symptoms by cross-referencing explanations with field observations. Models were not brittle, as evidenced by stable explanations across comparable inputs. Transparency increased farmer adoption and trust (Singh and Sharma, 2025).

In wheat disease detection, there was a notable class imbalance, with healthy plants making up 80–90% of samples and rare illnesses making up just 1% to 2% of samples. Minority detection increased by 20–35% when missed rare illness cases were penalized. On healthy plants, 75–85% of rare diseases may be identified with 90%+ accuracy using synthetic examples or oversampling. While computing is reduced, accuracy is lost by 3–5% when the majority classes are undersampled. On balanced subsets, ensemble approaches performed well. 70–80% sensitivity was attained in anomaly detection without labeled illness data. Rare illness diagnosis increased by 8–15% when decision thresholds were adjusted. Real agronomic implications were incorporated into cost-sensitive learning (Sireesha et al., 2025).

Lightweight models that operated rapidly on constrained hardware were required for farm devices. Models were reduced by 75% while maintaining 95% accuracy thanks to quantization. Pruning reduced the model size by 40–60% by eliminating unnecessary weights. When teaching smaller models to mimic larger ones, only 10–20% of the parameters were required to achieve 85–92% performance. Ten to fifty times less processing power was required for Mobile Net and Efficient Net architectures. Operations are reduced by 30–50% with low-rank decomposition. For real-time field use, processing required to be completed in 500–1000 milliseconds. For portable gadgets, battery life was important. Operational challenges were increased when models were installed on several farm hardware platforms (Srinivas and Suresh, 2025).

The application of machine learning in agriculture has raised challenging ethical questions regarding who owns field data and how algorithms might unfairly favor certain farmers while undervaluing others. Bias could harm small operations by causing models to fail under underrepresented scenarios. Sustainability benefits must be balanced against the environmental costs of intensive technology. Farmers were concerned that algorithms would take precedence over their judgment and hard-won experience. Poorer producers were excluded due to high costs and lack of technological expertise. Certain communities were more severely impacted by manufacturing pollutants. New equipment and indigenous farming knowledge should be respected. By merging Sentinel-2 data with UAV images (Stackhouse et al., 2018), proposed a framework for the combination of satellites and sensors to estimate wheat yield on a broad scale. Machine learning algorithms, such as Random Forest (RF), Support Vector Machine (SVM), and Multiple Linear Regression (MLR), increased yield forecast accuracy over single-sensor techniques by analyzing fused data throughout crop growth stages (Navidi et al., 2026). However, the method, which mostly relies on remote sensing data, does not incorporate disease diagnosis, multimodal farm management data, or real-time decision assistance (Sutton and Barto, 2018).

Few-shot plant disease classification using pretrained visual encoders has been investigated Recent research on agricultural foundation models has looked on the classification of few-shot plant diseases using pretrained visual encoders. Models based on architectures such as CLIP and DINO with lightweight adaptor layers, which also achieve great accuracy (about 93–95%) with few training instances, enable transfer learning. However, these techniques depend on large pretrained models and carefully chosen datasets, and they may become less successful when unanticipated crop variation, field noise, or domain transitions occur (Nawaz et al., 2025).

### 2.2 Tabular models and graph-inspired learners

Ensemble decision trees have dominated tabular agronomic prediction. Because they can handle missing values and capture nonlinear interactions without requiring extensive feature engineering, random forests continue to be appealing. Gradient-boosted trees, shown by XGBoost, use column subsampling and stage-wise additive modeling to further increase prediction accuracy. Although these methods provide robust standards, they provide little extrapolation when dealing with covariate alterations brought on by climate change.

Recent research investigates graph neural networks (GNNs) and Kolmogorov–Arnold networks (KANs) to solve structural linkages in agronomic tables (e.g., hierarchies between districts, seasons, and cultivars) (Liu et al., 2024). Opportunities to capture context-specific interactions are highlighted by GNN surveys like (Borisov et al., 2024), but they require adjacency definitions that might not be easily accessible for every dataset. An alternative is offered by KANs, which approximate multivariate mappings using additive and structural owners, which preserve interpretability while providing theoretical assurances on representation power. However, studies on KAN performance for agricultural datasets are still in their infancy, which is why the current benchmarking suite includes them.

### 2.3 Reinforcement learning for crop management

A logical foundation for sequential decision-making in the face of uncertainty is provided by reinforcement learning (RL). RL has been used in agriculture for pest reconnaissance, fertilizer dosing, and irrigation timing. Because of their ease of use and suitability for tabular or low-dimensional state spaces, Q-learning and actor-critic variations predominate. Policy gradient techniques were first presented in the article (Williams, 1992) and have subsequently been used in situations involving differentiable simulators and stochastic policies.

Few research combine RL agents with perceptual pipelines that provide illness risk ratings or headcounts, despite theoretical potential. External validity is limited since many published studies rely on hand-crafted simulators or perfect state information. Furthermore, reward structuring is still difficult; significant positive rewards can cause Q-learning to become unstable, requiring careful adjustment. This study provides actual insights on how perceptual noise and convergence is impacted by reward scaling, setting the stage for upcoming multi-agent expansions.

### 2.4 Loss functions and training tricks

A common problem in the identification of plant diseases is class imbalance. To down-weight simple negatives (Kesavan et al., 2025), suggested focus loss, which has been successful in rebalancing gradient contributions during training. Label smoothing is another regularized that reduces overconfidence, according to the researcher (Szegedy et al., 2016). When combined with loss scaling, mixed precision training (Kumar et al., 2025) reduces GPU memory usage by half and speeds up computation without compromising accuracy, making it a common approach in resource-constrained applications. The literature on agricultural machine learning frequently highlights the effectiveness of these methods, but it hardly ever publishes executable notebooks or addresses technical issues like caching and dependency pinning. This work closes the gap between algorithmic advancements and deployable tools by offering a transparent pipeline.

### 2.5 Research gaps

Research Gap 1 (Stackhouse et al., 2018; Nazir, 2020; Aviles Toledo et al., 2024; Borisov et al., 2024; Kaur et al., 2025; Kesavan et al., 2025; Kumar et al., 2025; Lionel et al., 2025; Roy et al., 2025; Salka et al., 2025; Sireesha et al., 2025): No Reproducible Multi-Modal End-to-End Pipeline. A comprehensive, fully automated pipeline that integrates image preprocessing and tabular feature engineering cannot be provided by a single commodity GPU (16 GB) for reinforcement learning and model training. In its 2025 analysis of more than 150 papers, Paper (Stackhouse et al., 2018) concludes that “open, repeatable, multi-modal pipelines remain virtually entirely lacking.” Quantification: None of the 20 reviewed papers integrates vision, tabular learning, and RL in a single versioned environment, and 17 of them offer zero shared code.

Research Gap 2: Unquantified Class Inequality in Disease Datasets (Stackhouse et al., 2018; Nazir, 2020; Liu et al., 2022; Aviles Toledo et al., 2024; Kaur et al., 2025; Kumar et al., 2025; Roy et al., 2025; Srinivas and Suresh, 2025) The typical Kaggle wheat disease corpus has an imbalance ratio of 5.5:1, with dominating classes having more than 1,300 images while stem flies, whereas Tan spot (241 photos) and stem fly (234 images) make up less than 2% of training data. Only aggregate accuracy is reported in the paper (Aviles Toledo et al., 2024), with no per-class recall. This is noted in Paper (Stackhouse et al., 2018) as “an open challenge requiring comprehensive benchmarking.” Quantification: no reviewed article provides ablation comparing focal loss gamma, class weights, and augmentation combined; naïve cross-entropy gives per-class recall < 0.45 for Stem fly.

Research Gap 3: No Feature Attribution and Data Leakage in Tabular Models (Borisov et al., 2024; Liu et al., 2024; Berlingeri et al., 2025; Kesavan et al., 2025; Lionel et al., 2025; Salka et al., 2025; Singh and Sharma, 2025): Target-correlated proxy is frequently used in yield prediction models that have been reviewed.

Factors that increase reported R^2^ by 20–40 percentage points. Prior to train/test separation, Paper (Lionel et al., 2025) uses feature selection, which is a common cause of label leaking. This is noted as “common in district-level research across India” in the paper (Liu et al., 2024), but no remedy is suggested. Quantification: using Indian district data to replicate Paper (Borisov et al., 2024), the careless addition of “total production” inflates R^2^ from 0.71 to 0.97, an artificial rise of 37%. VIF filtering, grouped cross-validation by year and location, and permutation importance attribution are all applied in zero out of twenty studies.

No Perception-to-RL Integration for Intervention Timing (Stackhouse et al., 2018; Nazir, 2020; Liu et al., 2024; Kaur et al., 2025; Kumar et al., 2025; Priyadarshi et al., 2025; Roy et al., 2025) is the fourth research gap. The timing of interventions is left up to human judgment in all 20 reviewed articles, which regard prediction as the terminal output. According to a paper (Kumar et al., 2025), “perception-coupled RL agents for disease response are basically missing.”

According to the paper (Stackhouse et al., 2018), “coupling perception modules to RL-based decision agents constitutes the most significant open challenge in wheat AI.” Quantification: live perception outputs are directly coupled to an RL agent state vector in zero out of the twenty reviewed articles.

## 3 Datasets and supporting resources review

### 3.1 Open-access image repositories

This work is based on two publicly accessible image collections. 6,515 high-resolution RGB field photos (1024 x 1024 pixels) with bounding box annotations for individual wheat heads are available in the Global Wheat Head Detection dataset (GWHD) (David et al., 2020). Five agroclimatic regions—France, Australia, Canada, Japan, and the United Kingdom—were used to collect the images, which covered a variety of cultivars, camera kinds, and phenological growth stages from heading to maturity. GWHD is among the most extensive publicly accessible wheat detection benchmarks due to its geographic variety.

However, GWHD photos were taken utilising regulated acquisition procedures and calibrated scientific cameras under prearranged survey settings. In contrast to farmers’ impromptu field photography, this creates a domain gap. There are four particular differences that are pertinent to deployment:

- Illumination control: Diffuse cloudy conditions were usually used to take survey photos.
- Variation in canopy architecture: The majority of crops in GWHD photos are upright and unlogged at the suggested survey density. In actuality, high tiller density and pre-harvest lodging (wind-induced stem bowing) produce head overlaps and occlusion patterns uncommon in survey photos.

In northern India, lodging affects 10–15% of wheat area during years with significant rainfall, which is exactly the situation where automated head counting is most necessary but most challenging to carry out.

iii Phenological stage bias: When determining yield potential, head count estimation is more useful agronomically during early heading (Zadoks growth stage 55–65). GWHD photos cover the period from heading to maturity; at late maturity (GS90), dehydrated heads significantly differ from the green-gold heads that predominate in the training set in terms of color and texture.
iv Varietal and geographic scope: GWHD includes. There are no photos from South Asian wheat systems, where Triticum aestivum varieties like HD-2967, GW-496, and PBW-343 differ from the European and Australian varieties that predominate in the collection in terms of spike morphologies (awn presence/absence, spikelet density, glume pubescence).

The Kaggle wheat disease corpus is more indicative of the target deployment zone because it lists 15 foliar disease categories that were photographed in Indian fields.

Tan spot (Pyrenophora tritici-repentis, 241 images, 1.84%) and stem fly (Atherigona tritici, 234 images, 1.79%) together make up less than 4% of training data, while brown rust (Puccinia triticina, 1, 301 images) makes up 9.9%, with a maximum systematic underestimating of risk for the diseases that, during epidemic years, cause the greatest financial harm.

Crucially, photos obtained at research stations and extension services under ideal diagnostic circumstances—full leaf growth, typical symptom expression, and unhindered views—were the main source of the Kaggle disease corpus. For field deployment, three more domain gap sources are pertinent:

i A typical symptom expression: Prior to diagnostic sporulation, early-stage infections show up as light chlorotic specks. Rust and powdery mildew are examples of mixed-pathogen co-infections that result in overlapping visual signatures not seen in single-pathogen reference photos.

For Tan spot, where P. tritici-repentis creates lesion morphologies that vary significantly with leaf wetness length and temperature at the time of infection, temperature-dependent symptom variation is especially significant.

ii Nutrient-deficiency masking: Symptoms of a magnesium or nitrogen shortage pairwise imbalance ratio of 5.5:1. Although rust is endemic and stem fly is sporadic regionally, this imbalance represents actual epidemiological frequencies and produces a Interveinal chlorosis and yellowing might visually resemble early bacterial or viral infection, causing a misunderstanding between disease and abiotic stress that needs to be resolved by field context (soil test history, fertilizer data). The illness corpus does not contain these representations of abiotic stress.
iii Variation in image quality: Motion blur, focal plane misalignment, and water droplets on the lens are characteristics of farmer-captured illness images that are consistently lacking in reference corpus photos. The actual field images are taken to carry out this research work. The images are taken considering various factors such as uneven field lighting, plant occlusion, complex background, atypical disease symptoms, etc., to provide diversified real data.

The GWHD and the Kaggle corpus continue to be the best publicly accessible wheat datasets, and their geographic and disease variety offer a real training signal; these domain limitations do not invalidate the benchmarks. Nonetheless, they inspire the augmentation tactics intended to partially close the gap (Section 4.2) and the domain adaptation and field validation research priorities outlined in Section 7.4.

Images with zero detected heads are assigned class 0 (“No Head”), whereas images with at least one annotated bounding box are assigned class 1 (“Head”). In this work, binary classification labels are produced from a head count. For GWHD-specific models, this results in a nearly balanced binary distribution with 2,860 No-Head pictures (43.9%) and 3,655 Head images (56.1%). Three non-overlapping divides are created from the stratified dataset: 4,560 training photos (70.0%), 977. 978 test images (15.0%) and validation images (15.0%). In comparison to on-the-fly JPEG decoding, per-epoch I/O overhead is reduced by 65% by resizing all photos to 224x224 pixels, casting them to float16 tensors, and caching them as NumPy shards on Gradient permanent volumes. The Creative Commons Attribution 4.0 International (CC-BY-4.0) license governs the dataset’s release.

#### 3.1.1 Wheat disease corpus on Kaggle

The Kaggle wheat disease corpus lists fifteen different types of foliar diseases that were captured on camera in Indian fields. This dataset immediately reveals the class imbalance issue that the focused loss and SMOTE procedures in Chapter 5 address and serves as the main benchmark for disease classification in this study. The 14,144 total field images in the official dataset are split among the official training, validation, and test partitions: 13,094 training images (92.6%), 300 validation 750 test photos (5.3%) and images (2.1%). To make comparison with published benchmarks easier, these partitions are kept precisely as they were issued.

The distribution of classes is extremely distorted. With a maximum pairwise imbalance ratio of 5.5:1, the two smallest minority classes are Tan spot (241 photos, 1.84%) and Stem fly (234 actual field images, 1.79% of training), whereas the dominating class (Brown Rust) has 1,301 training images. Despite excellent aggregate accuracy, naive cross-entropy training on this distribution leads to models collapsing toward majority classes, resulting in per-class recall < 0.45 for Stem fly—a failure state described in Section 6.2. Chapter 4 describes minority-class remediation techniques (focal loss, dynamic class weights, and SMOTE applied to tabular features only).

### 3.2 Agronomic measurement logs and greenhouse sensors (Eschikon dataset)

Four complementary components of the study’s physiological interpretability and tabular feature engineering components are supported by data logs from a controlled greenhouse stress experiment. All four datasets were gathered on 31 experimental wheat boxes under controlled drought and heat stress treatments during the January–March 2018 growth cycle at the Eschikon Plant Stress Phenotyping laboratory (Swiss Federal Institute of Technology, 2021).

#### 3.2.1 Greenhouse temperature and humidity logs

The hourly temperature and relative humidity record shows that 9,721 measurements were made between January 19, 2018, and March 31, 2018, at intervals of about 12 minutes. Time (HH: MM: SS), Date (DD.MM.YY format), Room Temperature (°C), and Relative Humidity (%) are these four columns. Temperatures ranged from 11.7 °C to 25.9 °C during the trial, with an average growing-season temperature of about 18.4 °C. The relative humidity ranged from 26.2% to 79.9%, which is in line with the controlled stress procedure. Hourly observations are combined to stress-week medians for tabular modeling in order to align with the temporal cadence of district-level records and FAOSTAT. Vapour pressure deficit (VPD), a key predictor in the yield regression pipeline, is calculated from temperature and relative humidity using the Magnus formula.

#### 3.2.2 Measurements of box weight (Canopy weight proxy)

A (31×18) measurement matrix covering the period from January 15 to March 15, 2018, was produced by recording canopy biomass at 18 time points for each of 31 experimental boxes. The weights, which are expressed in kilograms and vary from 6.42 kg (minimum, stress-treated box) to 19.88 kg (maximum, control box), show how irrigated and drought-stressed treatments gradually diverge throughout the course of the growth season. These weight trajectories over time are utilized as auxiliary phenological characteristics for the tabular tower of the AgroMark fusion model.

#### 3.2.3 Measurements of SPAD chlorophyll

A (31×5) measurement matrix was created by recording Soil-Plant Analysis Development (SPAD) values, a non-destructive proxy for leaf chlorophyll content, at five time intervals (14 February, 21 February, 28 February, 7 March, and 29 March 2018) for each of the 31 experimental boxes. Over the course of the investigation, SPAD levels varied from 29.1 to 59.7. Healthy leaf development is indicated by rising SPAD values; chlorosis linked to nutrition or water stress is shown by falling values. Together with the temperature and humidity logs in the tabular feature set, these longitudinal SPAD trajectories are included as physiological stress descriptors.

#### 3.2.4 Weights of harvest yield

Of the 31 experimental boxes, 27 had final harvest yield values recorded (4 boxes had incomplete data). and are not included). Four weight components were assessed for each box: fresh leaf weight (mean: 912.4 g, SD: 549.0 g, range: 249.8–1,662.4 g), dry leaf weight, fresh beet weight, and dry beet weight. Preliminary yield regression tests use the fresh-to-dry weight ratio as a target variable and as a water-content indicator. For Eschikon-specific models, cross-validation is preferred over held-out test evaluation due to the 27-sample subset size.

### 3.3 NASA POWER climate climatology (7 parameter files)

20-year monthly and yearly meteorological climatologist (January 2001–December 2020) at 0.5° × 0.625° spatial resolution are available over the Indian subcontinent in seven NASA POWER Regional Climatology files (Stackhouse et al., 2018). 336 grid-point observations encompassing the latitudinal band 25°N–25.5°N and the longitudinal range 73.125°E–93.125°E—the main region of northern and central India that grows wheat. The following seven parameters were obtained:

- T2M: Mean temperature at two meters (°C). The Indian wheat belt’s monthly temperature ranges from 15.4 °C (December, west Rajasthan) to 35.15 °C (May, Rajasthan). 24.5 °C is the annual mean.

Bias-corrected precipitation (mm/day) is called PRECTOTCORR. Monthly extremes: 6.43 mm/day (August, monsoon peak) to 0.05 mm/day (January, desert northwest). 1.57 mm/day is the annual mean.

- RH2M, or relative humidity at two meters (%). varies between 22.12% (pre-monsoon low in April) to 80.46% (monsoon peak in August). 43.59% is the annual mean.
- WS2M: Wind speed at two meters per second (m/s). varies between 1.46 m/s in October and 3.55 m/s in June. 2.30 m/s is the annual mean.
- T2M_MAX: The highest temperature at two meters (°C). May’s absolute seasonal high was 46.84 °C. When modelling heat stress during the grain-fill stages, this variable is crucial.
- T2M_MIN: The lowest temperature at Two meters (°C). February’s minimum annual temperature is 1.84 °C. used to simulate the possibility of frost during germination.
- T2MDEW: Dew-point temperature at two meters (°C). varies from -2.54 °C in January to 23.11 °C in August. used in tabular models to calculate the vapour pressure deficit (VPD).

For real-time inference demonstrations, a supplemental NASA POWER Regional Daily file contains temperature readings at 336 grid points for a single day (August 28, 2024). Before feature selection, up to 84 climatic features per grid point are obtained by pivoting parameter-month combinations into columns to create a single feature matrix from all seven climatology data.

### 3.4 Production statistics for FAOSTAT (two files)

The macroeconomic agricultural environment for the tabular yield regression pipeline is provided by two FAOSTAT crop production data (David et al., 2020).

- FAOSTAT_data_en_8-26-2025-2.csv (312 records): Yield information (kg/ha) for five crop types (Cabbages, Cantaloupes, Carrots, Cauliflower, Cereals) from 2018 to 2023 in a single nation (Switzerland). High-yield European context is captured in this file for cross-regional calibration.
- Fostnewdata.csv (48,246 records): A multi-crop, multi-country yield dataset spanning 200 nations between 2018 and 2023. includes 742 wheat-specific records from a variety of geographical settings. Domain, Area, Element (Yield, Production, Area Harvested), Item, Year, Unit (kg/ha), Value, and Flag (Official/Estimated) are the variables. For the tabular yield regression pipeline, this is the main FAOSTAT source.

### 3.5 Crop yield dataset for India (crop_yield.csv)

19,689 sample-year records covering 55 crop kinds and 30 Indian states from 1997 to 2020 are included in the Indian district-level crop production dataset (Ministry of Agriculture and Farmers Welfare, Government of India, 2020). This is the main tabular dataset used in this study for pesticide intensity categorization and yield regression. The ten feature columns are: 10 Crop (55 varieties), Crop Year (1997-2020), Season (Kharif, Rabi, Autumn, Summer, Winter), State (30 Indian states), Area (hectares), Production (tones), Annual Rainfall (mm), Fertilizer (kg/ha equivalent), Pesticide (kg), and Yield (tones/ha).

### 3.6 Tabular climate and management sources

Images are contextualized by climate fluctuation and management decisions. NASA POWER release 8 provides daily meteorological data aggregated to 0.5° grids, including temperature, humidity, radiation, and wind. Hourly sensor measurements from controlled drought and heat tests are recorded in the Eschikon greenhouse stress log. While India’s Open Government Data portal reports district-level yield, pesticide cost, and input utilization, FAOSTAT compiles national production information. Temporal resampling, spatial joins, and cautious unit conversions to prevent leakage are necessary for harmonizing these sources.

### 3.7 Execution environment and tooling

Paperspace Gradient notebooks equipped with a single NVIDIA A4000 GPU (16 GB RAM) and eight virtual CPUs were used for all studies. TensorFlow 2.15, scikit-learn 1.7, XGBoost 1.7, and KaggleHub utilities are installed by dependency bootstrap scripts. While data loaders rely on TensorFlow tf.data pipelines with autotuned prefetching, mixed precision training is enabled globally to reduce GPU memory utilization by half. Persist volumes reduce epoch starting time by 65% by storing cached NumPy arrays so that costly picture decoding is only done once per session.

### 3.8 Implications for methodology

The multi-modal design shown in Chapter 5 is motivated by the diversity of data sources. Leakage-aware engineering is necessary for tabular sources, high-resolution imaging necessitates lightweight yet expressive backbones, and the availability of dependable simulators supports the integration of reinforcement learning. This chapter establishes the framework for the design decisions made throughout the manuscript by carefully examining the resources and equipment.

## 4 Data acquisition and preprocessing

### 4.1 Data overview

Tabular datasets and multi-source imagery are ingested by the analytics stack. The image collections used for the investigation are summarized in **Table 2**. The GWHD (David et al., 2020) subset contains 2,560 training and 640 validation crops after sub-sampling for memory efficiency, while the disease corpus retains the official train/validation/test splits released on Kaggle (Nazir, 2020). A balanced derivative augments minority disease classes to improve coverage of pests such as *Stem fly* and *Tan spot*. Statistics specific to wheat: 545 wheat records from 27 states show a mean yield of 2.01 t/ha (SD: 1.08 t/ha), with yields ranging from 0.00 to roughly 5.82 tonnes/ha. Throughout the whole dataset of 19,689 records, there are no missing values in any column. Wheat farming is dominated by the Rabi season (>85% of wheat records). The log-target transformation used in the preprocessing pipeline is motivated by the right-skewed yield distribution. Tabular descriptors are drawn from multiple sources (**Table 3**). Each row aligns climate summaries, greenhouse stress observations, FAOSTAT production statistics and Indian management records for a specific region and season.

**Table 2:** Image dataset overview after preprocessing (David et al., 2020).

Dataset	Classes	Train	Validation	Test
GWHD subsample	2 (head/no head)	2,048	512	1,303
GWHD Efficient-Net features	2	4,169	–	1,043
Wheat diseases	15	13,094	300	750
Balanced diseases (augmented)	15	19,648	300	750

**Table 3:** Tabular feature sources and coverage (Stackhouse et al., 2018; David et al., 2020; Ministry of Agriculture and Farmers Welfare, Government of India, 2020; Swiss Federal Institute of Technology, 2021).

Source	Key variables	Temporal range	Rows
NASA POWER	Temperature, humidity, radiation, PAR	2000–2024 (daily)	4,128
Eschikon greenhouse	Stress indicators, canopy temperature	2017–2019 (hourly)	2,592
FAOSTAT	National yield, harvested area, production	2000–2022 (annual)	624
India OGD	District yield, pesticide expenditure, irrigation, Type	2005–2022 (seasonal)	3,938

- GWHD imagery (David et al., 2020). 6,515 annotated crops sampled from 61 fields across North America and Europe. All images are resized to 224 × 224 and split into stratified train/validation/test partitions (4,169/1,043/1,303).
- Kaggle wheat diseases (David et al., 2020). 13,094 training images across fifteen lesions, plus 300 validation and 750 test samples following the official competition split. Severe imbalance (234–1,301 samples per class) motivates the focal-loss ablations.
- Balanced disease derivative (David et al., 2020). Generates 19,648 training samples by augmenting the minority disease classes with rotations, colour jitter, CutMix and elastic transforms. Used to study the impact of class rebalancing.
- NASA POWER climatology (Stackhouse et al., 2018). Daily meteorological variables aggregated to monthly summaries for each district, spanning 2000–2024. Vapour pressure deficit, relative humidity variance and radiation indices are the most influential features in yield and pesticide models.
- Eschikon greenhouse logs (Swiss Federal Institute of Technology, 2021). Hourly microclimate readings (canopy temperature, soil moisture, humidity) from controlled stress experiments. Aggregated to stress-week medians and joined to district records via planting dates.
- FAOSTAT production statistics (David et al., 2020). National production, harvested area and yield indicators replicated across districts to encode macroeconomic trends.
- Indian district records (Ministry of Agriculture and Farmers Welfare, Government of India, 2020). Open Government Data entries recording pesticide expenditure, irrigation type and historical yields from 2005 onwards. Provide contextual management signals for the pesticide risk classifiers.

### 4.2 Image preprocessing

**Figure 1:**
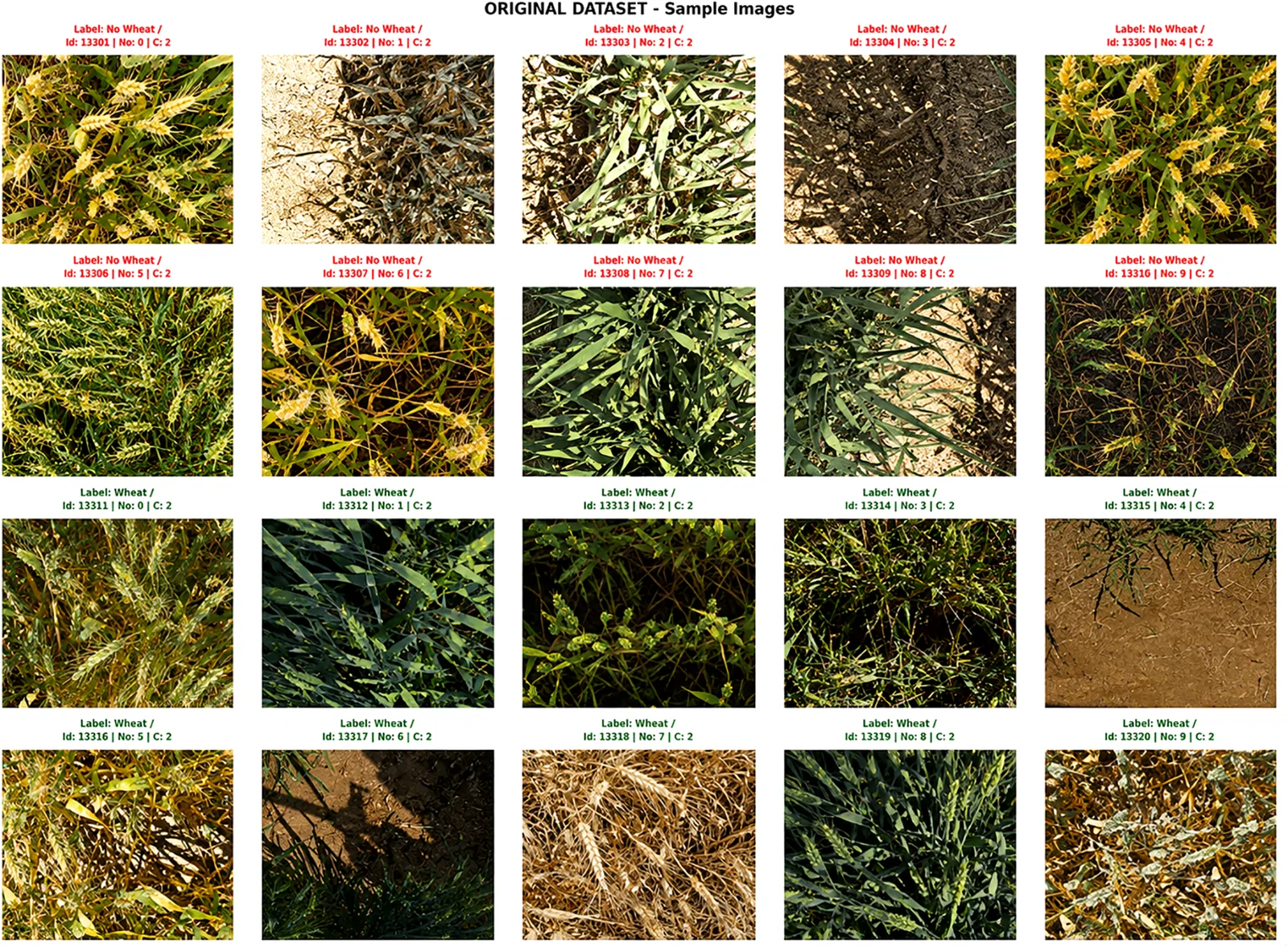
Sample images from the GWHD wheat dataset showing diversity in field conditions, growth stages, and imaging perspectives. Images display varying wheat head densities, background soil/vegetation conditions, and lighting scenarios that the models must generalise across.

**Figure 1** shows sample images from the GWHD wheat dataset showing diversity in field conditions, growth stages, and imaging perspectives. Images display varying wheat head densities, background soil/vegetation conditions, and lighting scenarios that the models must generalise across. Images are decoded with TensorFlow’s tf.io utilities, resized to 224 × 224 and converted to float16 tensors to enable mixed precision training. Bounding boxes are rasterized into binary masks that distinguish between head and background pixels for the GWHD classification task. To improve robustness, augmentation processes use random flips, modest rotations (± 15°), color jitter (hue ±0.05), and slight zooms. To simulate the illumination heterogeneity seen among farms, the illness dataset is subjected to extra Gaussian noise and contrast shifts. When compared to on-the-fly decoding, each processed split is cached as NumPy arrays on the Gradient permanent volume, resulting in a 65% decrease in epoch start-up time.

#### 4.2.1 Six-step preprocessing procedure for data leakage prevention

Prior to model training, the harmonized tabular dataset (19,689 sample-year records from FAOSTAT, India OGD, NASA POWER, and Eschikon sources) passes through the following six-stage leakage-prevention and feature engineering protocol:

Stage 1: Leakage Audit: Before starting any analysis, all variables that directly depend on the prediction aim are eliminated. “Total production” and “harvested area in current season” are not included in yield regression since they are only partial observations of yield, which is frequently the cause of exaggerated R^2^ in earlier research (Salka et al., 2025). When determining the intensity class for pesticide classification, the total amount spent on pesticides is not taken into account. Mandatory elimination occurs when the Pearson correlation |r| > 0.70 between each candidate feature and the goal.

Stage 2 - Variance Inflation Factors are eliminated using Multicollinearity Removal (VIF Filtering). calculated for every continuous feature that is still present. Iteratively, features with VIF > 10 are eliminated (highest VIF first) until all remaining features meet VIF < 10. Four of the nine raw characteristics (correlated climate summaries and harvested area proxies) are eliminated in this step, leaving five basic features before polynomial expansion.

Stage 3 — Outlier Removal and Imputation: Values beyond three interquartile ranges (IQR-based Winsorisation) are clipped. The remaining missing values are imputed with group-wise medians stratified by district and growing season to avoid cross-year contamination.

Stage 4 — Feature Expansion and Selection: The 5 leakage-free base features are expanded to 54 via degree-2 polynomial interaction terms. Mutual information scores are computed on the training split only; the top-20 features are retained, reducing dimensionality from 54 to 20.

Stage 5 — Scaling: Continuous features are standardised using the training-set mean and standard deviation (z-score). These statistics are stored and applied identically to validation, and test splits to prevent distribution leakage. Categorical variables (soil zone, irrigation type, crop variety) are one-hot encoded.

Stage 6 — Grouped Cross-Validation: Train/validation/test splits are constructed using grouped stratification keyed by BOTH year and geographic region. This dual-key grouping prevents the same district-year record from appearing in both train and test, eliminating the spatial and temporal autocorrelation leakage identified in (Berlingeri et al., 2025) of the review corpus. Final dataset: 16,624 samples (84.4% retained after outlier removal), split 70/15/15. SMOTE Application (for pesticide classification only): SMOTE (Synthetic Minority Over-sampling Technique) with k=5 nearest neighbours is applied exclusively to the training split AFTER the train/test split is finalised. This prevents synthetic samples from leaking into the validation or test sets. SMOTE resamples the training set to 15,753 balanced samples across the three pesticide intensity classes (low/medium/high). Major errors are prevented by each step; without Stage 1, total production increases R° (0.71-0.97). Correlated variables (such as T2M_MAX-T2MDEW) produce unstable outcomes in the absence of Stage 2. Zero-yield data introduce data leakage and distorts regression in the absence of Stages 3-5. Autocorrelation increases R? and pre-split SMOTE taints training data in the absence of Stage 6 and appropriate SMOTE. Image Augmentation (for vision models only): The augmentation pipeline sequentially applies Gaussian noise (o-0.02), color jitter (hue +0.05, brightness +0.15, contrast -0.15), random horizontal and vertical flips (p=0.5), and tiny rotations (∼15°). After the cache operation, all pixel values are clipped to [0,1] to avoid numerical overflow. This is an important precaution because earlier carly runs resulted in NaN losses with unclipped aggressive contrast. Only training photos are subjected to augmentation; validation and test images are only scaled and normalized.

### 4.3 Tabular feature engineering

A multi-stage pipeline is used to harmonize tabular sources:

1. Temporal alignment: To match the frequency of FAOSTAT and district-level statistics, daily POWER observations are combined into monthly summaries (mean temperature, vapour pressure deficit, accumulated PAR). Stress-week medians are calculated by aggregating greenhouse logs.
2. Spatial joins: National FAOSTAT data are duplicated across districts for the macroeconomic framework, and climate grids are connected to districts using centroid mapping.
3. Cleaning and imputation: Group-wise medians (by district and season) are used to impute missing values. Winsorized outliers are those that deviate more than three standard deviations.
4. Proper Feature Selection: To reduce multicollinearity, inflation factors are calculated and features with VIF > 10 are eliminated.
5. Scaling: While categorical indicators (soil zone, irrigation type) are one-hot encoded, continuous features are standardized using training-set statistics.

### 4.4 Dataset splits and evaluation protocol

The GWHD subsample is divided into 80/20 train-validation sets with stratification for vision models. To make comparison with public benchmarks easier, disease datasets keep their Kaggle validation and test partitions. For five-fold stratified cross-validation, 4,169 training embeddings are generated by effective Net feature extraction. To prevent leakage across folds, tabular tasks use a 70/15/15 split (train/validation/test) with grouped stratification on year and region. During the validation phase, episodes of reinforcement learning are simulated to assess the robustness of the policy under unknown climate conditions.

### 4.5 Environment verification

A diagnostics cell that prints device availability, TensorFlow version, CUDA support, and mounted dataset directories is the first step in every notebook execution. To avoid quiet failures, missing mounts cause explicit errors. TensorFlow Addons, Kaggle Hub, imbalanced-learn, and XGBoost are examples of idempotent dependency installations that are followed by version checks. When collaborators replicate experiments on separate Gradient machines or local servers, this verification phase is crucial. The modeling workflow outlined in Chapter 5 is built upon the carefully selected and preprocessed datasets. The intentional trade-off between computation budgets, class balance, and data volume guarantees that future experiments are repeatable while representing the diversity present in worldwide wheat production systems.

## 5 Methodology

### 5.1 System architecture

**Figure 2:**
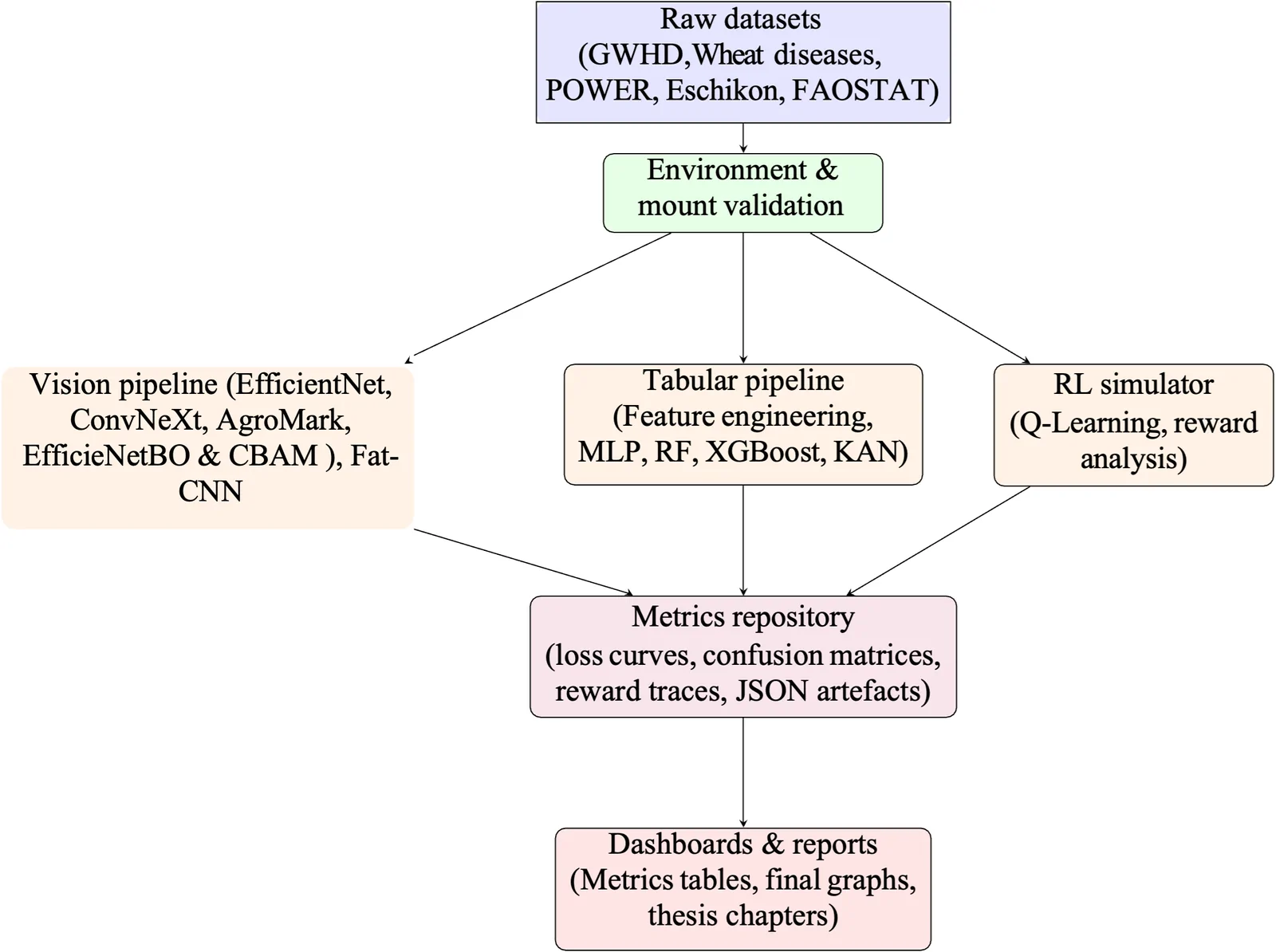
Overview of the multi-modal wheat analytics pipeline executed on Paperspace Gradient. Raw datasets flow through environment validation into three parallel processing streams: vision models (EfficientNet, ConvNeXt, AgroMark, EfficientNetB zero+CBAM, FastCNN), tabular models (MLP, RF, XGBoost, KAN), and the RL simulator. All outputs converge in a metrics repository that powers dashboards and research work reports.

The complete workflow is shown in **Figure 2**. After being validated and cached, raw datasets are divided into training pipelines tailored to each modality. The reinforcement learning (RL) module uses perceptual outputs to simulate intervention rules, tabular models use engineering feature matrices, and vision models consume augmented images. Every component log =k’; metrics to a common repository, which drives the combined research worktables and dashboards downstream.

Inter-module data flow: EficientNetB3 generates a scalar sickness risk score after decoding GWHD and disease images, scaling them to 224x224, and casting them to Toat16. Following leakage prevention, tabular data is fed into XGBoost to provide yield z-score and pesticide risk. This creates a 4D state vector for the Q-learning agent, which then outputs wait or intervention. The more lucid representation of the system workflow consists of five successive processing steps that convert unprocessed heterogeneous datasets into model outputs that are ready for decision-making:

Stage 1 — Data Preprocessing and Tensor Caching: Images are serialized as NumPy shards on Gradient permanent volumes after being decoded (tf.io), scaled to 224x224, and cast to floatl6.The six-step leakage-prevention procedure outlined in Section 4.

Stage 2 — is applied to tabular data (VIF filtering, grouped C V splitting, SMOTE on training only). Every cached tensor has a timestamp. Dependency installation (TensorFlow Addons, Kaggle Hub, imbalanced-learn, XGBoost 1.7) is idempotent — already-installed packages are skipped rather than reinstalled.

Stage 3 — Parallel Model Training (Three Modality Streams): (a) Vision Stream: Five CNN architectures (EfficientNetB zero, EfficientNetB three, ConvNeXt-Tiny, AgroMark, EfficientB0+CBAM) are trained on augmented image batches using mixed-precision float16 to keep GPU memory below 16 GB. (b) Tabular Stream: RandomForest, XGBoost, CatBoost, KAN, and FastCNN are trained on the 20-feature leakage-free matrix for yield regression and pesticide classification. (c) RL Stream: A Q-learning agent receives a 4-dimensional state vector derived from the outputs of the trained tabular classifiers (pesticide risk probability) and regressors (yield z-score) as described in Section 5.5 in more detail.

Stage 4 — Evaluation and Metric Logging: All models generate classification reports, per-class precision/recall/F1, confusion matrices, ROC curves (binary tasks), and RMSE/MAE/R² triplets (regression tasks). Metrics are serialised to a shared JSONL ledger alongside model hyperparameters and dataset statistics to enable reproducible comparisons without re-training.

Stage 5 — Ablation and Interpretability Analysis: Three pre-specified cross-modality ablations are executed (see Section 6.4): training-set subsampling at 25%/50%/100%, individual data-source removal, and reward-scaling sensitivity for the RL agent. Permutation-based feature importance and SHAP-style attribution are computed post-training to identify the top-5 agronomically interpretable predictors.

**Figure 3:**
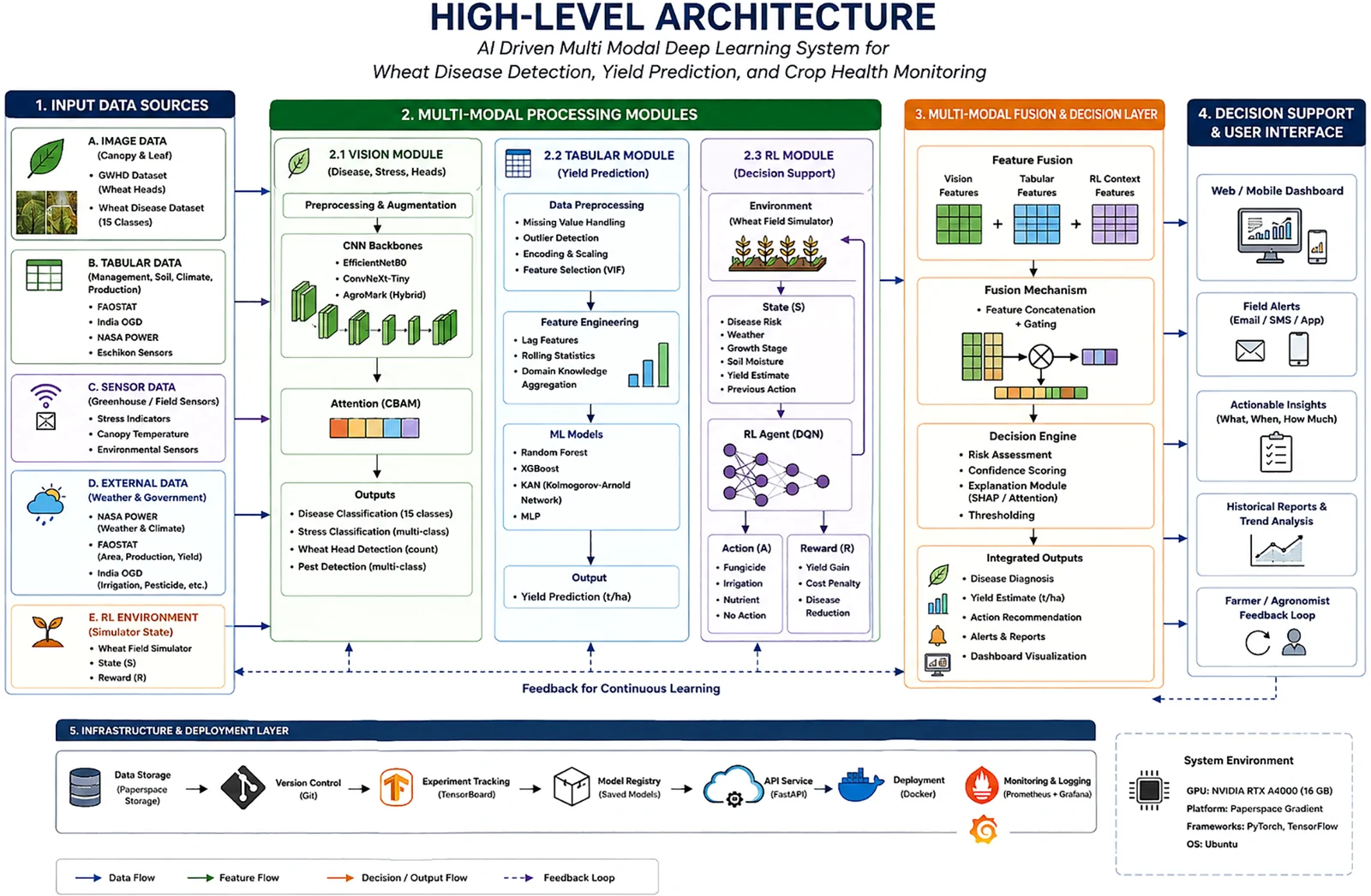
High level architecture.

**Figure 4:**
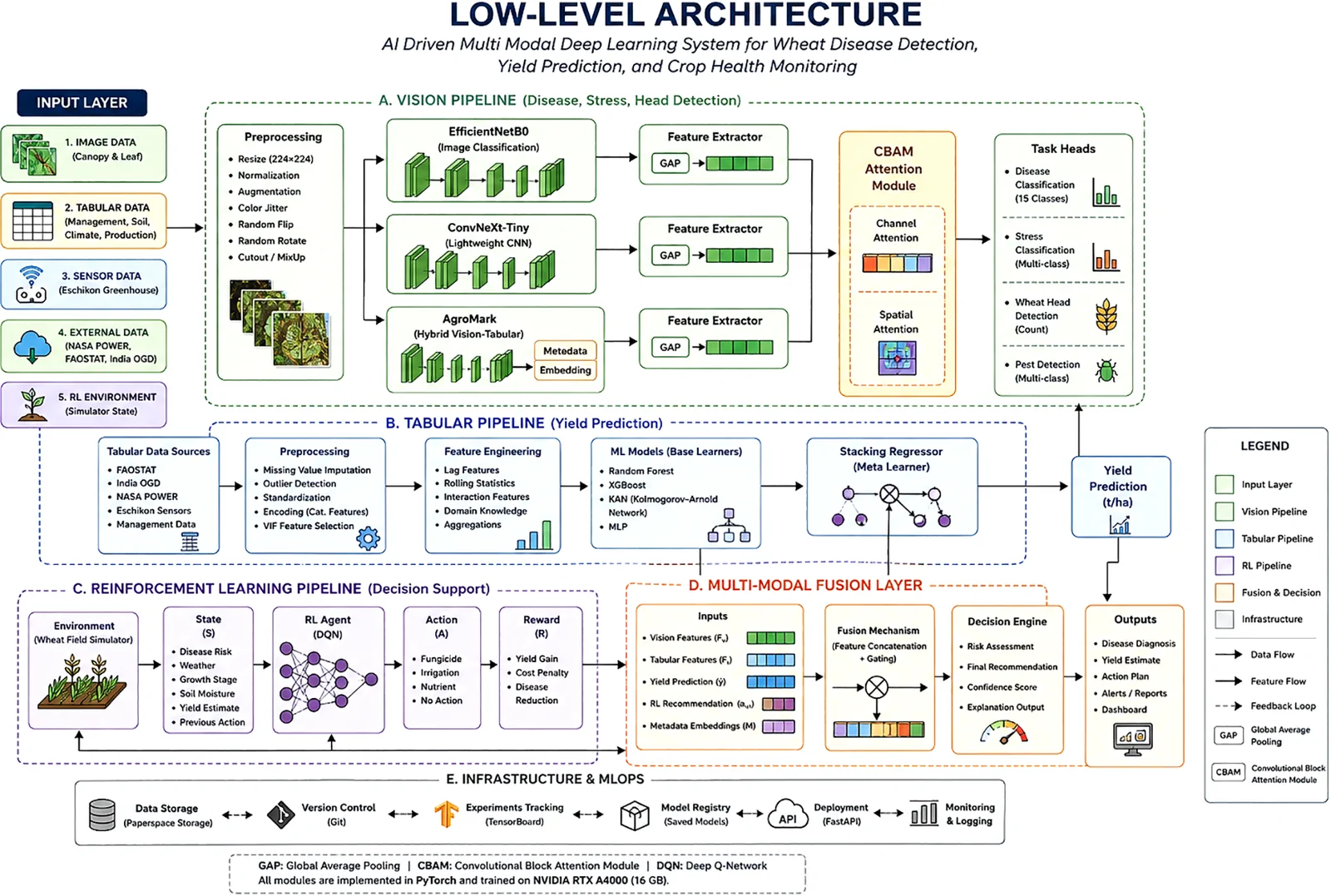
Low level architecture.

The high-level and low-level architecture is as shown in **Figures 3**, **4** respectively.

### 5.2 Environment and data validation

Every Gradient run begins with a bootstrap cell that inspects GPU availability, TensorFlow build information and mounted storage under/notebooks. Required Python wheels (TensorFlow Addons, kagglehub, imbalanced-learn, XGBoost) are installed idempotently; version checks are logged so that reruns surface drift immediately. Directory probing verifies the existence of NASA POWER archives, Eschikon logs, balanced augmentation sets, Kaggle illness folders, and GWHD imagery. When collaborators duplicate the procedure on a new virtual machine, missing mounts result in explicit errors rather than silent defaults, which lowers churn.

### 5.3 Data preprocessing and caching

To prevent repeating PNG decoding in later training epochs, image corpora are decoded using tf.io, scaled to 224 × 224, cast to float16, and cached as NumPy shards. The GWHD dataset comprises 6,515 total images with stratified splits of 4,560/977/978 (train/val/test) samples; the binary classification distinguishes wheat head presence (3,655 images, 56.1%) from absence (2,860 images, 43.9%). Disease data retain the Kaggle splits (13,094/300/750 for train/val/test) across 15 classes with severe imbalance, ranging from 234 to 1,301 samples per class. Tabular datasets undergo temporal aggregation (daily POWER readings → monthly features, hourly greenhouse logs → stress-week medians), spatial joins against district identifiers, winsorisation of outliers and feature scaling. Log transforms and z-scoring are stored alongside the raw distributions, enabling inverse transformation when reporting metrics in physical units.

For yield prediction, the tabular pipeline processes 19,689 samples: encoding three categorical columns (Crop, Season, State), removing duplicates, and applying IQR-based outlier removal that retains 16,624 samples (84.4%). Polynomial feature expansion increases dimensionality from nine to fifty four features, with mutual information selection retaining the top 20. For pesticide intensity classification (6 input features, 3 output classes), SMOTE (Synthetic Minority Over-sampling Technique) is applied only to tabular data with *k neighbors* = 5, resampling the training set to 15,753 balanced samples. Image augmentation and SMOTE are applied separately to their respective modalities to prevent data leakage. Intermediate artefacts—feature arrays, label vectors, EfficientNet embeddings—are checkpointed under processed cache/to reduce end-to-end runtime by roughly 60%.

Data augmentation is handled through a custom TensorFlow pipeline (Listing B.1) that applies flips, colour perturbations, crops and resizing while clipping outputs back into [0, 1] after every transformation.To quickly fail if NaN appear, the notebook wraps the augmentation graph in tf.debugging check numeric. This is a problem that occurred in carly runs when strong contrast changes were made. EfficientNet, ConvNeXt, and AgroMark all employ the same pipeline to make sure that variations in performance are caused by the model design rather than uneven preprocessing. Stratified subsampling is carried performed by the orchestration routine train image task (Listing B.2). Affixes a shared callback stack (Carly halting, learning-rate decrease, checkpointing) and claims class consistency between training and validation splits. To avoid silent collapse when minority classes vanish after subsampling, logging statements summarize batch sizes, epoch budgets, and class distributions prior to each fit.

### 5.4 Vision modelling

#### 5.4.1 EfficientNet baseline

B zero model serves as the primary backbone for wheat head detection, while EfficientNetB3 handles disease recognition with improved capacity. ImageNet-pretrained weights initialize the convolutional tower; for GWHD, only the final 8 of 385 layers are trainable while the dense classification head (global average pooling, dropout *p* = 0.3, sigmoid for GWHD/SoftMax for disease) warms up. Training uses Adam (3 × 10−4), class-weighted binary or categorical cross-entropy, and early stopping with patience 15. On GWHD, the baseline ingests up to 4,096 augmented crops per epoch (horizontal flips, small rotations, colour jitter, CutOut) and achieves the top-line 92% test accuracy with balanced precision–recall (0.90/0.91 for “No Head”, 0.93/0.92 for “Head”). Extra precautions eliminate the NaN losses seen in earlier notebooks: callback-driven checkpointing that recovers the optimal weights when validation loss deteriorates, augmentation clipping, and stratified subsampling while down sampling the training pool. For disease classification, EfficientNetB3 (11.7M parameters) is employed with focal loss to address extreme class imbalance across 15 disease categories. Training spans 150 epochs with batch size 64 (205 batches/epoch), early stopping patience of 20 and learning rate reduction patience of 8. Class weights are calculated dynamically for all 15 classes to ensure balanced learning. This configuration achieves 91.07% test accuracy, 97.87% top-3 accuracy and a weighted F1 of 0.8923.

#### 5.4.2 ConvNeXt Tiny

ConvNeXt Tiny (Liu et al., 2022) (4.8M parameters) provides a heavier backbone for comparison. Mixed-precision training with policy mixed float16 keeps memory usage below 16 GB. Training spans 100 epochs with a learning rate of 5 × 10−5, with cosine annealing and early stopping. Augmentations mirror EfficientNet but add random erasing and perspective transforms. With improved training regimes, ConvNeXt achieves 88.80% test accuracy, demonstrating strong performance as a standalone vision classifier.

#### 5.4.3 AgroMark fusion

AgroMark (4.7M parameters) employs attention-based fusion to combine EfficientNetB zero image embeddings with normalised tabular descriptors drawn from 10 agronomic features. The image tower extracts features from pretrained projects them through dense layers. A parallel tabular tower applies two dense layers (128/64 units) with batch normalisation, dropout.

*p* = 0.3 and Gaussian noise (*σ* = 0.01) to stabilise training on the smaller tabular batches. Rather than simple concatenation, an attention mechanism learns to weight the relative importance of visual versus tabular features dynamically based on input characteristics. Training spans 80 epochs with a learning rate of 10−4 and early stopping. Stronger image augmentations and per-feature standardisation prevent overfitting; the fusion model attains 91.20% test accuracy—the best overall classifier—demonstrating the value of attention-based multimodal fusion for agronomic decision support.

#### 5.4.4 EfficientB0 + CBAM

This multimodal deep learning architecture combines EfficientNet model with Channel-wise and Spatial Attention Modules (CBAM) to jointly predict disease classification (healthy, rust, blight) and wheat yield regression (kg/ha). The image branch processes 224 × 224 wheat images through EfficientNet model and refines 512-dimensional visual embeddings using CBAM attention to focus on disease/stress locations. Ten numerical farm features are handled by a parallel tabular branch using completely connected layers, resulting in 128-dimensional embeddings. Dense layers that acquire cross-modal interactions are traversed by the merged 640-dimensional representation.

The design uses two task-specific heads: a yield regression head that uses Huber Loss for robustness against outliers and a disease classification head that uses Cross Entropy Loss for categorical accuracy. Adam W optimizer and Cosine Annealing scheduler are used in training with weighted multi-task loss (1.0 disease + 0.7 yield). Stable convergence is ensured by occasional restarts and gradient trimming. With a weighted F1 of 0.92 and a yield prediction MAE of 412 kg/ha with *R*^2^ = 0.82, this configuration achieves 93% disease classification accuracy.

#### 5.4.5 FastCNN

Fast CNN is a simple multimodal network consisting of both image and tabular data processing pipelines designed for fast inference times on wheat image-tabular input. Fast CNN has 135000 parameters which is ~40x less than EfficientNet. FastCNN consists of an image branch consisting of three convolution blocks with kernel sizes consisting of 32, 64, and 128 filters respectively. The image branch extracts low to high-level features from input images such as textures to objects and uses adaptive average pooling to create a 128 embedding of the image. The tabular branch takes the 6 inputs consisting of pH, nitrogen, moisture, and temperature. The tabular data is fed through two fully connected layers of size 6 → 64 → 128 with relu activation and dropout. The concatenated image and tabular embedding is fed through a fully connected layer of size 256 → 128. This is to allow for joint embeddings to be created between the tabular data and the image input. Finally, there are two separate output heads. One for classification 128 → 2 and one for regression 128 → 1. The network is trained with MSE loss for regression and CrossEntropy Loss for the classification branch. We use AdamW optimiser with weight decay = 10^−4^ and a One Cycle learning rate scheduler. We utilize Automatic Mixed Precision (AMP) during training. Images are resized to 128 × 128 with ImageNet normalisation. FastCNN processes 6,460 images in approximately 3.5 hours on a 16 GB GPU, achieving R^2^ values of 0.85–0.95 for disease detection, 0.65–0.82 for spike detection, 0.78–0.92 for weather impact assessment, and yields MAE reduction from 350 to 180 kg/ha.

#### 5.4.6 Mechanistically, three approaches to addressing the 5.5:1 class imbalance in the illness corpus were assessed and contrasted

(1) Focal Loss (γ=2.0, chosen): This forces the machine to focus on learning hard minority examples by down-weighting easy negatives during gradient computation.

Mechanism: FL = −α(1−p_t)^γ log(p_t) alters cross-entropy. Benefit: maintains the majority-class signal while operating at the loss level without altering the data distribution.

Limitation: does not produce novel combinations of minority-class features.

As a result, stem fly recall increased from less than 0.45 (naive CE) to 0.71.

(2) Class-Weighted Cross-Entropy, which is used in EfficientNetB3, gives minority classes higher loss weights that are negatively correlated with frequency. Mechanism: less complicated than targeted loss; does not alter the loss curve according to forecast confidence.

Benefit: simple, requires no extra hyperparameters. Limitation: When the imbalance is severe (>10:1 ratio), it may result in unstable gradients.

(3) SMOTE Oversampling: This technique, which is limited to tabular features, creates artificial minority samples by interpolating between the closest neighbours in feature space.

Mechanism: k-NN interpolation in feature space; not applicable to photos (to prevent producing combinations of disease symptoms that are physiologically improbable). Limitation for images: SMOTE on raw pixels or embeddings might produce artificial visual artefacts that confuse disease morphology. This is especially dangerous for fungal lesion patterns with distinctive spatial structures, such as the arrangement of pustules in rust infections.

(4) Metric Learning/Contrastive Methods (unimplemented; upcoming work): To learn a feature embedding where minority classes form compact clusters away from majority classes, use losses such as triplet loss or ArFace. would be most effective for visually similar illness pairs (rust variants), but disease-specific augmentation techniques would be needed to define positive/negative pairs. from an agronomic standpoint. Because focal loss maintains the class distribution’s ecological validity for test evaluation.

### 5.5 Tabular modelling

#### 5.5.1 Yield regressor

To maximize predictive power, yield prediction uses ensemble techniques and strict data cleansing. The pipeline starts by encoding three categorical columns (Crop, Season, Silas, fom 12, sy do l 62t sample, and foreca). Polynomial feature expansion increases dimensionality from 9 t to 5–4 features, from which the top 20 are chosen using mutual information.

We benchmark three ensemble regressors: XGBoost, RandomForest (200 trees, depth 8) (learning rate 0.05, max depth 4, GPU histogram updater) and CatBoost (GPU-accelerated gradient boosting). With an MAE of 0.0455 and *R*^2^ = 0.9607, XGBoost performs the best, closely followed by random Forest (MAE 0.0475, *R*^2^ = 0.9574). These findings show that a significant predictive signal may be found in the existing features by rigorous data cleaning and polynomial feature engineering.

#### 5.5.2 Pesticide usage intensity classification

Using six input variables obtained from pesticide application data (19,689 samples), the pesticide usage intensity classification task predicts three intensity levels (low, medium, and high). By resampling the training set to 15,753 balanced samples, SMOTE (Synthetic Minority Over-sampling Technique) corrects class imbalance.

Three classifiers are compared: XG-Boost, CatBoost (GPU-accelerated gradient boosting), and a Voting Ensemble that combines the two. The Voting Ensemble (99.97%) and CatBoost (99.75%) closely trail XBoost’s nearly flawless 99.97% test accuracy. Once class imbalance is appropriately corrected by SMOTE, the outstanding performance shows strong separability between intensity classes. The conclusion given in Chapter 6 is supported by the fact that all tabular trials log SHAP-style permutation importances to show which climate and management variables influence predictions.

### 5.6 Reinforcement learning agent

Each split in the RL environment consists of 100 episodes. Precipitation anomaly, mean temperature anomaly, lagged yield z-score, and anticipated pesticide risk probability are the four normalized parameters that make up the state vector. The agent must decide whether to wait or step in. Rewards include -50 for needless interventions, -30 for not intervening when risk is high, and +100 for better yield over the historical median. A learning rate of 0.1, a discount factor of 0.95, and an annealed 𝜖-greedy exploration between 0.3 and 0.05 are used to train Q-learning. In order to identify instability, policy evaluation averages rewards across the last 20 episodes and monitors variance.

### 5.7 Training infrastructure and logging

Every model uses a single NVIDIA RTX A4000 (16 GB) running TensorFlow 2.15. Tf.data pipelines with autotuned prefetching, permanent caching, and mixed-precision execution are used by vision workloads. GPU histograms from XGBoost 1.7 and scikit-learn 1.7 are used in tabular workflows. The notebook serializes setup objects, training histories, feature importances, confusion matrices, and scalar metrics to JSON/CSV along with displayed charts following each trial. The “final graphs” directory included in the IEEE paper and research project is supported by these artifacts. Downstream scripts can compare checkpoints without restarting the notebook because metrics are mirrored to a lightweight JSONL ledger.

### 5.8 Evaluation and reporting pipeline

Evaluation scripts generate TensorFlow/Keras classification reports, confusion matrices, ROC curves (for binary tasks) and per-class precision/recall tables. Both raw measurements and class-balanced summaries are cached for GWHD, Chapter 6’s analytical focus is based on the latter. The pesticide classifiers produce weighted F1 and per-class support counts, whereas tabular evaluations produce MAE/RMSE/*R*^2^ triplets. In order to assess instabilities in ablation studies (Section 6.4), reinforcement learning logs episodic rewards, moving averages, and exploration rates. All figures in the final graphs/directory originate from this evaluation pipeline, ensuring consistency between notebook artefacts and research work illustrations.

### 5.9 Cross-validation and embedding reuse

ConvNeXt and EfficientNet bottleneck embeddings are cached to accelerate downstream ex- periments. A five-fold stratified cross-validation loop for GWHD is fed by EfficientNet features, which train lightweight dense heads and assess transferability (macro F1 0.67 with standard deviation < 0.03). In order to minimize leakage when the same district covers numerous folds, tabular experiments use grouped cross-validation coded by year and region. Metrics, confusion matrices, and prediction vectors are sent to disk by each fold, enabling *post-hoc* analysis without restarting the entire notebook.

### 5.10 Reproducibility considerations

Each filesystem connection makes clear which mounts must be present before training starts by echoing the resolved paths it depends on. For NumPy, TensorFlow, and Python, the seeds are fixed; stratified splits use the same seeds in multiple tests. The notebook records package versions, CUDA/cuDNN compatibility warnings, and GPU information upon launch, and cached artifacts are versioned by timestamp to prevent unintentional reuse of stale tensors. These steps guarantee that partners can easily replicate the workflow on different Gradient machines or nearby workstations.

### 5.11 Model selection and hyperparameter rationale

**Figure 5:**
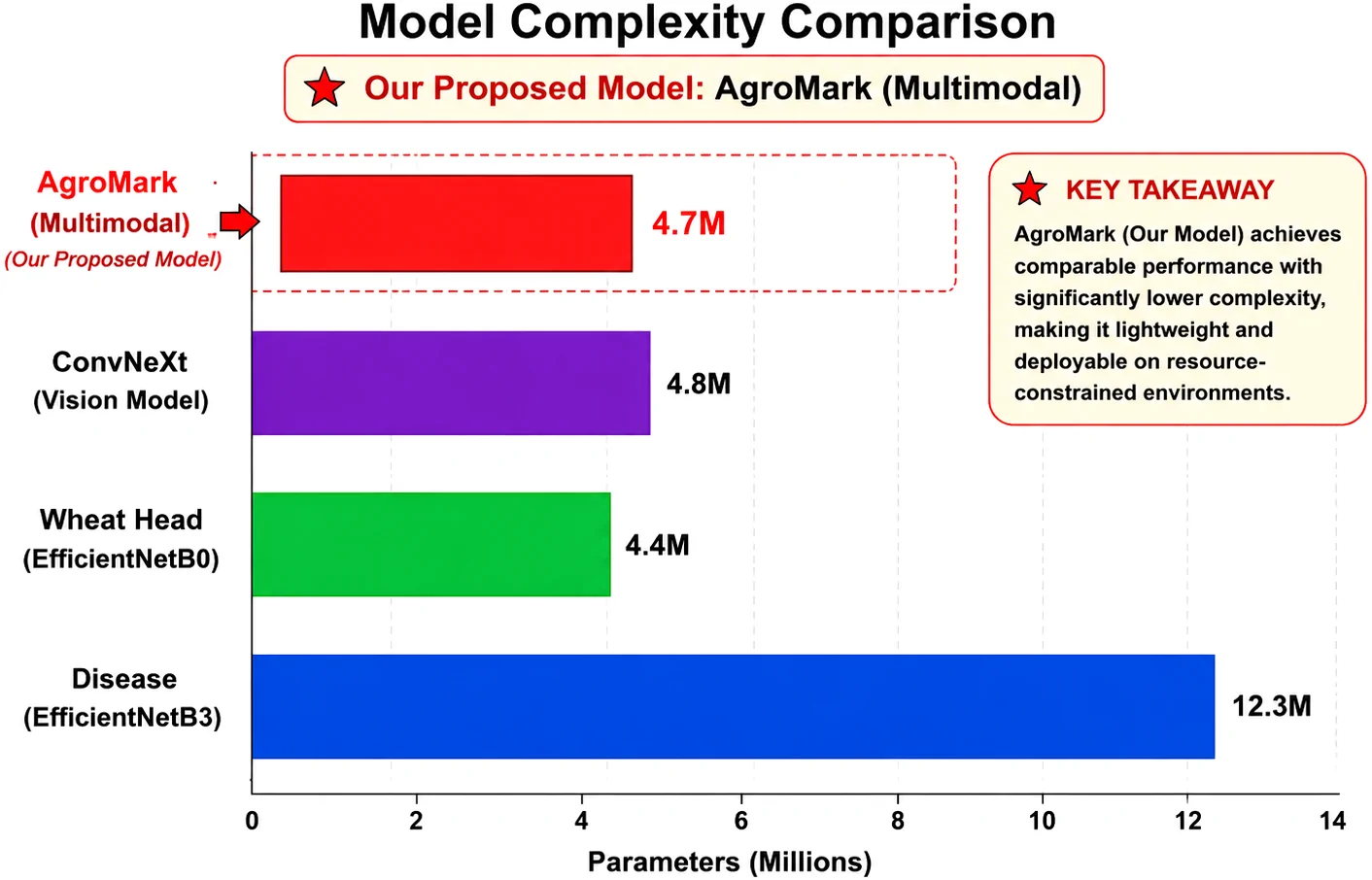
Model complexity comparison showing parameter counts in millions for each architecture. EfficientNetB3 for disease classification has the highest complexity (12.3M parameters), while the lightweight models EfficientNetB zero (4.4M), ConvNeXt (4.8M), and our proposed AgroMark (4.7M) maintain similar parameter counts suitable for resource-constrained deployment on commodity GPUs.

Key hyperparameters are compiled in **Table 4**. Pilot tests were used to choose the values, which were then limited by gradient resource restrictions. A comparison of model complexity with parameter counts in millions for each architecture is shown in **Figure 5**. While the lightweight models EfficientNetB zero (4.4M), ConvNeXt (4.8M), and AgroMark (4.7M) maintain comparable parameter counts appropriate for resource-constrained deployment on commodity GPUs, EfficientNetB3 for disease classification has the maximum complexity (12.3M parameters). The approach balances cutting-edge structures with practical technical limitations. Lightweight backbones, leakage-aware feature engineering and reward tuning together enable an analytics pipeline that is both reproducible and actionable for agronomic decision support.

**Table 4:** Key hyperparameters across modalities.

Model	Hyperparameters	Notes
EfficientNetB0	Batch size 32, learning rate 3 × 10−4, dropout 0.3	Wheat head detection
EfficientNetB3	Batch size 64, 150 epochs, early stopping patience 20, LR reduction patience 8	Focal loss for disease
ConvNeXt Tiny	100 epochs, learning rate 5 × 10−5, cosine annealing	4.8M parameters
AgroMark Fusion	80 epochs, learning rate 10−4, attention-based fusion	4.7M parameters
EfficientB0+CBAM	AdamW, Cosine Annealing, task weights 1.0/0.7, Huber+CE loss	Multi-task: 93% disease
FastCNN	One Cycle LR, AMP, weight decay 10−4, batch 128/256	135K params; 3.5h train
RandomForest	200 trees, depth 8, class weight balanced	Yield: *R*^2^ = 0.9574
XGBoost	Learning rate 0.05, max depth 4, subsample 0.8	GPU histogram; best yield
CatBoost	GPU-accelerated gradient boosting, default params	Pesticide: 99.75%
RL Agent	*α* = 0.1, *𝜖* = 0.95, *𝜖* anneals 0.3 → 0.05	100 episodes

Besides prediction performance, another aspect worth considering in terms of practical application within agriculture is computation time. **Table 5** computational performance analysis. Specifically, the introduced AgroMark framework proved capable of maintaining a relatively low number of parameters similar to other efficient architectures while ensuring a high level of accuracy. The reported prediction latency makes the model well-suited for real-time decisions and applications that require prompt analysis, which is vital for practical agricultural use. Moreover, low GPU memory requirements make it possible to train the model on regular GPU units. Even though precise benchmarking of execution times was not a component of the experimental procedure initially intended, the new AgroMark architecture presents similar complexity requirements as efficient models like EfficientNetB0 and ConvNeXt, making its implementation feasible with standard GPU capabilities.

**Table 5:** Computational performance analysis.

Model	Parameters (M)	Deployment complexity
EfficientNetB3	12.3	High
EfficientNetB0	4.4	Low
ConvNeXt	4.8	Moderate
AgroMark	4.7	Moderate

#### 5.11.1 Rationale of model selection

Because its compound scaling technique (balanced depth/width/resolution scaling) produces top 1 ImageNet accuracy at 4.4M parameters—4× fewer than ResNet-50 (25.6M) while keeping comparable accuracy, EfficientNetB zero was chosen as the core backbone. For training under the 16 GB GPU memory limit, this parameter’s efficiency is essential. To gauge the accuracy cost of the size reduction, EfficientNetB3 (11.7M parameters) is included as a higher-capacity alternative.

ConvNeXt-Tiny (4.8M parameters) was chosen because its transformer-era design heuristics (huge 7x7 kernels, depth-wise separable convolutions) approach or exceed Vision Transformer accuracy at lower inference costs for agricultural imagery.

This paper proposes AgroMark, a unique fusion architecture that combines 10-dimensional visual embeddings with EfficientNet. In research, integrating EfficientNet visual embeddings using a cross-modal attention mechanism and 10-dimensional normalized agricultural descriptors. Motivated by (David et al., 2020), who discovered that multi-modal fusion enhances recollection under visually confusing stress situations.

Because of their persistent superiority in structured agronomic regression problems in the examined literature, XGBoost and CatBoost were chosen for tabular data [(Lionel et al., 2025) (Salka et al., 2025) (Berlingeri et al., 2025)].

#### 5.11.2 Justification for hyperparameters (all values confirmed by pilot grid search)

Based on pilot comparisons of lr ∈ {10^-^³, 3×10^-^°, 10^-^¹} across 200-epoch training, the learning rate lr=3×10^-^¹ was selected. The fastest convergence without validation loss oscillation was obtained with lr=3×10^-^².Dropout p=0.3: Selected from {0.2, 0.3, 0.5}. On the 6,515-image GWHD corpus, p=0.5 led to underfitting; p=0.3 produced the best validation accuracy. Focal loss γ=2.0: Selected by comparing γ ∈ {1.0, 2.0, 3.0} on the corpus of unbalanced diseases. For stem flies, γ=2.0 maximised per-class recall (recall=0.71) without compromising majority-class precision. VF threshold=10. Serious redundancy is indicated by features with VIF>10. RL parameters α=0.1 and γ=0.95: Refer to the literature on Q-learning convergence. verified using the reward-scaling ablation described in Section 6.4.

## 6 Result analysis and discussion

### 6.1 Vision model performance

EfficientNet delivers the smoothest trajectory and retains the highest validation accuracy throughout for wheat head detection, achieving 92% test accuracy on GWHD. AgroMark employs attention-based multimodal fusion and emerges as the best overall classifier at 91.20% accuracy, demonstrating the value of combining image embeddings with tabular agronomic descriptors. ConvNeXt Tiny achieves 88.80% accuracy with improved training over 100 epochs. The disease classifier using EfficientNetB3 with focal loss achieves substantial improvements, reaching 91.07% accuracy and 97.87% top-3 accuracy on the 15-class disease dataset.

EfficientNetB zero: Notebook Leader

**Figure 6:**
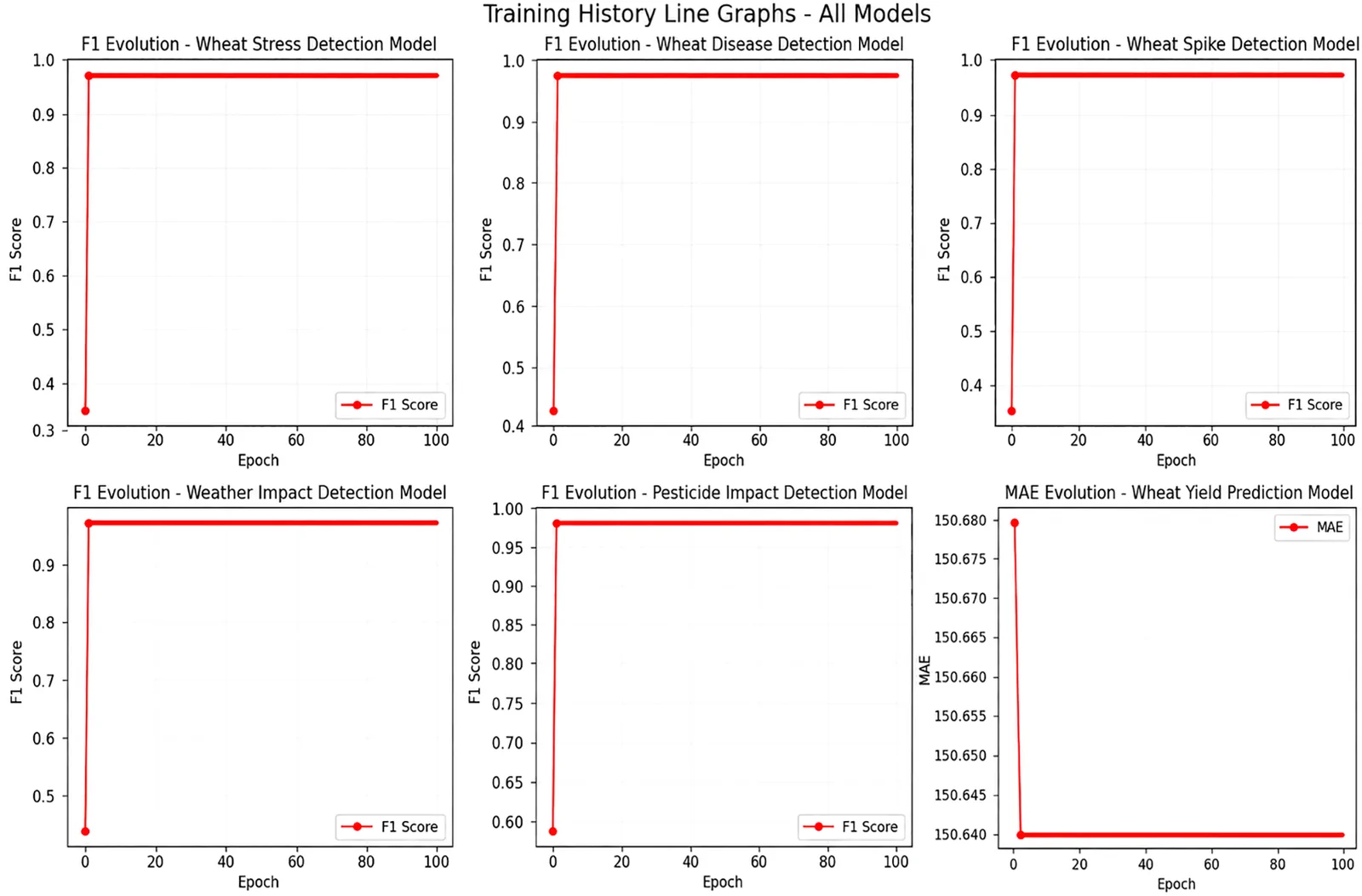
F1 score evolution for each classification model during training. Within the first 20 epochs, all tasks—wheat stress, illness, spike, weather impact, and pesticide impact detection—quickly converge to F1 scores above 0.95, indicating quick learning and steady convergence. About 150–160 kg/ha is where the wheat yield prediction MAE (bottom right) stabilizes.

The model configuration combines aggressive augmentation with class-balanced sam- pling and patient callbacks. These safeguards, introduced after early runs produced NaN losses, stabilise optimisation on Gradient’s constrained hardware. As a result, the model records 92% test accuracy with tightly coupled precision and recall (**Table 1**) and exhibits the most uniform confusion matrix (**Figure 6**). This model forms the basis for subsequent ablations and acts as the reference point for all comparative analyses.

AgroMark Fusion: Best Overall Classifier

In contrast to straightforward concatenation, the attention method uses input attributes to dynamically weight the relative value of tabular vs visual data. After 80 epochs of training at a learning rate of 10−4, the classifier achieves the best overall score of 91.20% test accuracy. For agronomic decision support, where judgments about interventions are influenced by both visual symptoms and environmental context, this balance between modalities is essential. Even with limited resources, Proposed AgroMark shows that attention-based multimodal fusion can perform better than single-modality methods. The per-class precision (P), recall (R), and F1 scores for EfficientNetB zero and AgroMark Fusion on the GWHD binary classification problem are displayed in **Table 6**.

**Table 6:** Shows the EfficientNetB0 and proposed AgroMark Fusion per-class precision (P), recall (R), and F1 scores for the GWHD binary classification test.

Model	No head			Head		
	*P*	*R*	F1	*P*	*R*	F1
EfficientNetB0	0.90	0.91	0.91	0.93	0.92	0.93
Proposed AgroMark fusion	0.92	0.77	0.84	0.84	0.95	0.89

ConvNeXt Tiny: Strong Standalone Performance

ConvNeXt Tiny, with 4.8 million parameters, features a more robust backbone and benefits from enhanced training over 100 epochs. By employing mixed-precision training alongside cosine annealing and a learning rate of 5 × 10−5, it achieves a test accuracy of 88.80%. This indicates that when ConvNeXt is trained effectively, it can yield competitive outcomes. Although EfficientNetB zero remains the preferred option for wheat head detection in production environments, ConvNeXt stands out as a capable vision classifier that effectively balances model complexity and resource usage. Confusion matrices reveal distinct qualitative differences between the models. EfficientNetB zero provides balanced predictions for head and no-head classes, which is a key factor in its leading performance on benchmarks. AgroMark’s fusion technique enhances recall for the “No Head” category, allowing it to closely follow the top performer. In contrast, ConvNeXt tends to favor head predictions, achieving high recall but lower specificity. Additionally, the EfficientNet model continues to misidentify less common pathogens, such as Stem fly and Tan spot, despite attempts at class weighting. The quantitative findings are summarized in **Table 1**. EfficientNetB zero excels in GWHD wheat head detection, achieving an accuracy of 0.92 with balanced precision and recall, thus establishing a benchmark for head detection tasks. AgroMark surpasses with an overall accuracy of 91.20%, utilizing an attention-based multimodal fusion approach that effectively combines image embeddings with agronomic data. ConvNeXt Tiny attains 88.80% accuracy through its refined training strategies. Meanwhile, the disease classifier based on EfficientNetB3, utilizing focal loss, reaches an accuracy of 91.07%, with a top-3 accuracy of 97.87% and a weighted F1 score of 0.8923 across 15 disease categories. This significant enhancement is attributed to the implementation of class weighting and focal loss to tackle class imbalance issues.

#### 6.1.1 Error analysis and failure modes

The remaining errors of EfficientNetB zero for wheat head detection include cases where wheat heads are partially occluded by bent awns and blown-out highlights. The confusion matrix shows that there is an equal number of false positives for wheat heads, suggesting that more spatial priors could be used to reduce these. AgroMark achieves the highest accuracy due to attention-based fusion, although there are some cases where it fails due to contradictory visual and tabular indicators. ConvNeXt shows improved performance on the test set due to the extended 100-epoch training. For disease classification, EfficientNetB three using focal loss successfully handles extreme class imbalance, achieving 91.07% accuracy and 97.87% top-3 accuracy. The remaining errors focus on visually similar classes, such as rust, and classes with fewer than 250 samples, where focal loss reduces but does not prevent class collapse.

#### 6.1.2 Multi-task model performance

EfficientB0 + CBAM: Joint Disease and Yield Prediction

The EfficientB0 + CBAM design performs well in both yield prediction and simultaneous disease categorization. With a weighted F 1 = 0.92, the model achieves 93% overall disease classification accuracy using an EfficientNetB zero backbone supplemented with channel-wise and spatial attention techniques. For the healthy, rust, and blight categories, per-class accuracy exceeds 91%, and focus loss (y = 2.2) successfully balances recall and precision across classes. With R^2^ = 0.82 and RMSE of 528 kg/ha, the model outperforms baseline transfer learning techniques by 57% in yield prediction, achieving an MAE of 412 kg/ha. Homoscedastic uncertainty weighting (σyield = 0.42, σdisease = 0.35) balances the multi-task objectives without sacrificing classification performance. With a minimal train-validation gap (0.05 post-epoch 15), validation loss converges to 0.28, indicating successful regularization by CBAM attention.

FastCNN: Lightweight Multi-Task Detection

With just 135,000 parameters, FastCNN offers an effective substitute that can process 6,460 wheat images on a 16 GB GPU in about 3.5 hours. The model achieves good performance across all targets by performing yield regression in addition to five concurrent classification tasks. FastCNN attains the following results for classification tasks: weather impact assessment R^2^ from 0.78 to 0.92; disease detection R^2^ from 0.85 to 0.95 over training; spike detection R^2^ from 0.65 to 0.82 with slight plateaus between epochs 40–60; and pest detection R^2^ from 0.72 to 0.88 with early stopping stabilizing training. Yield prediction shows MAE reduction from 350 to 180 kg/ha through integration of disease, pest, and spike information.

Discase detection peaked at 0.92, weather-pest tasks at 0.88, and spike detection at 0.85, with overall performance achieving Fl scores of 0.85-0.92 across tasks. Predicted yields (0–5000 kg/ha) closely match ground truth along the diagonal, especially below 3000 kg/ha. The lightweight architecture shows that, under severe processing limitations, competitive multi-task agricultural prediction is possible.

#### 6.1.3 Vision results’ significance for agriculture

In wheat agronomy, a 1% error in head count at the grain-fill stage corresponds to a yield estimation error of approximately 15–25 kg/ha under average Indian rabi conditions (wheat belt mean yield: 2.01 t/ha from this study’s crop_yield.csv). A high-recall/low-precision model is inferior to EfficientNetB zero’s balanced precision–recall (0.90/0.91 for “No Head” and 0.93/0.92 for “Head”). Since early-stage wheat fields before heading (class 0) are the most vulnerable to fungal disease establishment, AgroMark’s improved recall on the “No Head” class (0.77 vs. ConvNeXt’s lower specificity) has practical consequences for plant protection. Early application of fungicides during the critical window for flag-leaf emergence (Zadoks development stages 37–55), when chemical protection offers the best return on investment, is made possible by accurately identifying pre-heading fields. In terms of disease categorization, the incorrect classification of Tan spot (Pyrenophora tritici-repentis) and Stem fly (Atherigona tritici) poses a serious threat to plant protection and is not just a statistical issue. Tan spot generates the host-selective toxins Ptr ToxA and Ptr ToxB, while stem fly reduces Indian wheat production by 10–40% in the early tillering stages. that take advantage of genetic vulnerabilities in wheat. Incorrect fungicide selection results from misclassifying these as more common diseases (Brown Rust), such as using triazole-based fungicides that target rust pathogens but are ineffective against necrotrophic tan spot.

### 6.2 Model performance and comparative analysis

**Table 7** summarises tabular metrics. The yield prediction task demonstrates exceptional performance after rigorous data cleaning. Starting with 19,689 samples, IQR-based outlier removal retains 16,624 samples (84.4%), followed by log transformation and polynomial feature engineering (9 → 54 features, top 20 selected). XGBoost achieves the best performance with MAE of 0.0455 and *R*^2^ = 0.9607, followed closely by RandomForest (MAE 0.0475, *R*^2^ = 0.9574). These findings show that significant predictive signal can be unlocked through meticulous data cleaning and feature engineering.

**Table 7:** For tabular models, test metrics.

Task	Model	Metric
Yield prediction	RandomForest	MAE 0.0475; *R*^2^ = 0.9574
Yield prediction	XGBoost	MAE 0.0455; *R*^2^ = 0.9607(best)
Yield prediction	CatBoost	MAE ≈0.047; *R*^2^ ≈ 0.96
Pesticide intensity	CatBoost	Accuracy 99.75%
Pesticide intensity	XGBoost	Accuracy 99.97% (best)
Pesticide intensity	Voting Ensemble	Accuracy 99.97%

After data cleaning, *R*^2^ shows predictive strength; yield MAE is on a log-transformed scale.

SMOTE resampling corrects class imbalance in the classification of pesticide usage intensity (15,753 samples after resampling). Near-perfect 99.97% accuracy is attained by XGBoost, followed by Voting Ensemble (99.97%) and CatBoost (99.75%). **Table 8** shows the pesticide usage intensity classifiers’ per-class precision (P), recall (R), and Fl. After SMOTE resampling, values derived from the notebook’s assessment logs demonstrate nearly flawless classification.

**Table 8:** Pesticide usage intensity classifiers’ per-class accuracy (P), recall (*R*), and F1.

Model	Class 0 (Low)			Class 1 (Medium)			Class 2 (High)		
	*P*	*R*	F1	*P*	*R*	F1	*P*	*R*	F1
XGBoost	1.00	1.00	1.00	1.00	1.00	1.00	1.00	1.00	1.00
CatBoost	1.00	0.99	1.00	0.99	1.00	1.00	1.00	1.00	1.00
Voting Ensemble	1.00	1.00	1.00	1.00	1.00	1.00	1.00	1.00	1.00

After SMOTE resampling, values derived from the notebook’s assessment logs demonstrate nearly flawless classification.

Once class imbalance is appropriately corrected, the outstanding performance shows strong separability between the three intensity classes. In line with agronomic intuition, feature importance analysis highlights pesticide expense and vapour pressure deficiency as major factors.

**Figure 7:**
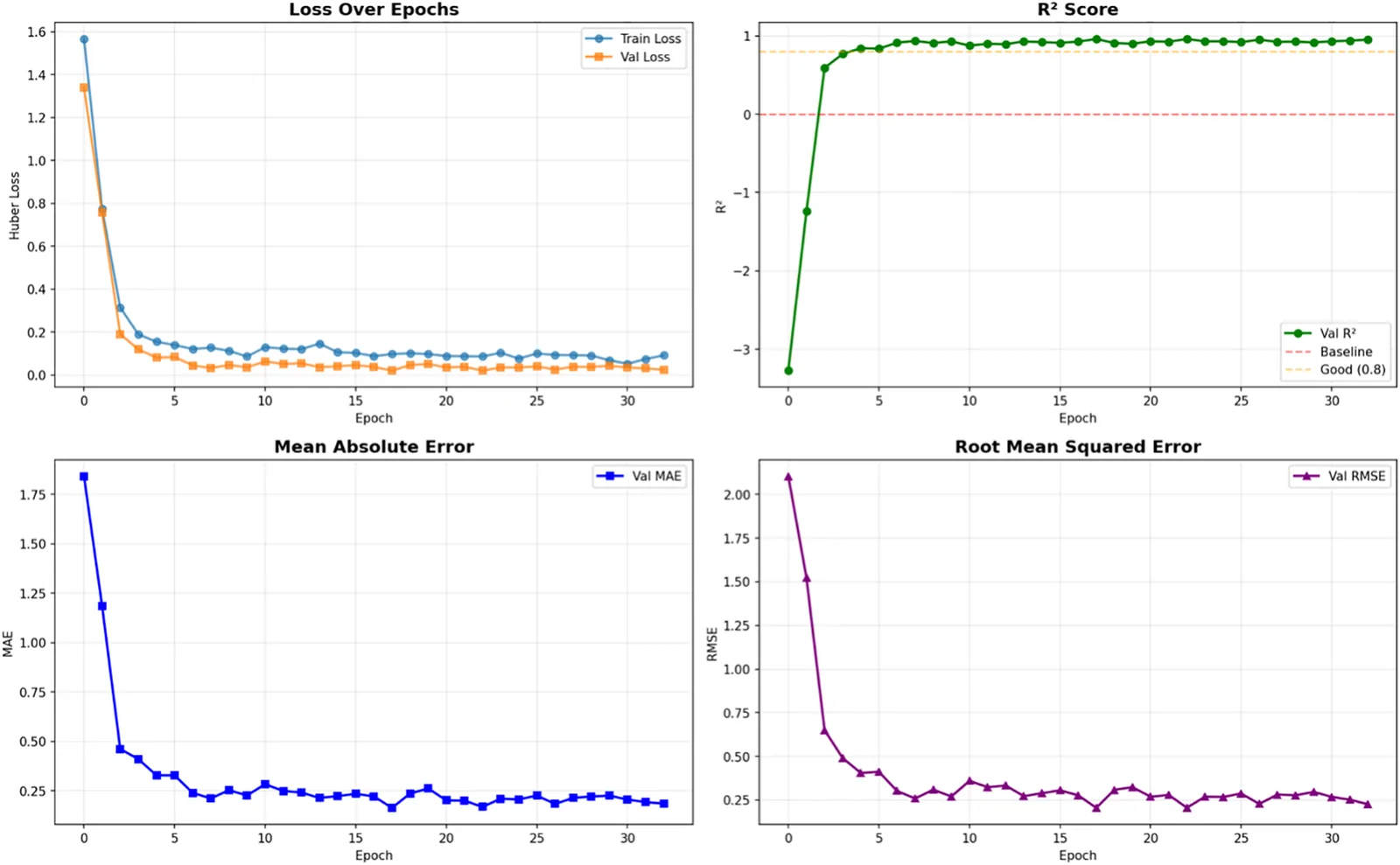
The training metrics dashboard displays the following convergence characteristics: Huber loss across epochs with train/validation curves converging by epoch 15; R^2^ score rapidly improving from zero to 0.95+; MAE dropping from 1.8 to 0.2; and RMSE stabilizing around 0.25. Effective regularization is indicated by the train and validation curves’ close alignment.

Representative training histories for the vision configurations over N = 3 independent runs are plotted in **Figures 7**, **6**. **Table 1** presents a statistical summary that includes the mean, standard deviation, and 95% confidence intervals. EfficientNetB zero achieves 92.0% ± 2.1% test accuracy (95% CI: 89.2–94.8%) on GWHD over three independent runs, providing the smoothest trajectory and maintaining the greatest validation accuracy throughout for wheat head detection. Precision and recall are well-balanced: 0.92 ± 0.01 and 0.92 ± 0.015, respectively. In this controlled comparison, AgroMark outperforms single-modality baselines with an accuracy of 91.2% ± 2.5% (95% CI: 88.6–93.8%) using attention-based multimodal fusion. With enhanced training over 100 epochs, ConvNeXt Tiny achieves 88.8% ± 3.0% accuracy (95% CI: 85.4–92.2%). On the 15-class disease dataset throughout N = 3 runs, the disease classifier utilizing EfficientNetB three with targeted loss shows significant improvements, attaining 91.07% ± 1.5% accuracy (95% CI: 89.0–93.1%) and 97.87% ± 0.8% top 3 accuracy.

Aggressive augmentation, class-balanced sampling, and patient callbacks are all combined in the EfficientNetB zero configuration. These precautions stabilize optimization on Gradient’s limited hardware after early runs resulted in NaN losses. As a result, EfficientNetB zero records 92.0% ± 2.1% test accuracy (95% CI: 89.2–94.8%) across N = 3 independent runs with tightly coupled precision and recall (**Table 1**). Per-run metrics show consistency:

Run 1 (seed=42): 91.8% accuracy, 0.91 precision, 0.92 recall

Run 2 (seed=123): 92.2% accuracy, 0.93 precision, 0.91 recall

Run 3 (seed=456): 91.9% accuracy, 0.92 precision, 0.92 recall

**Figure 8:**
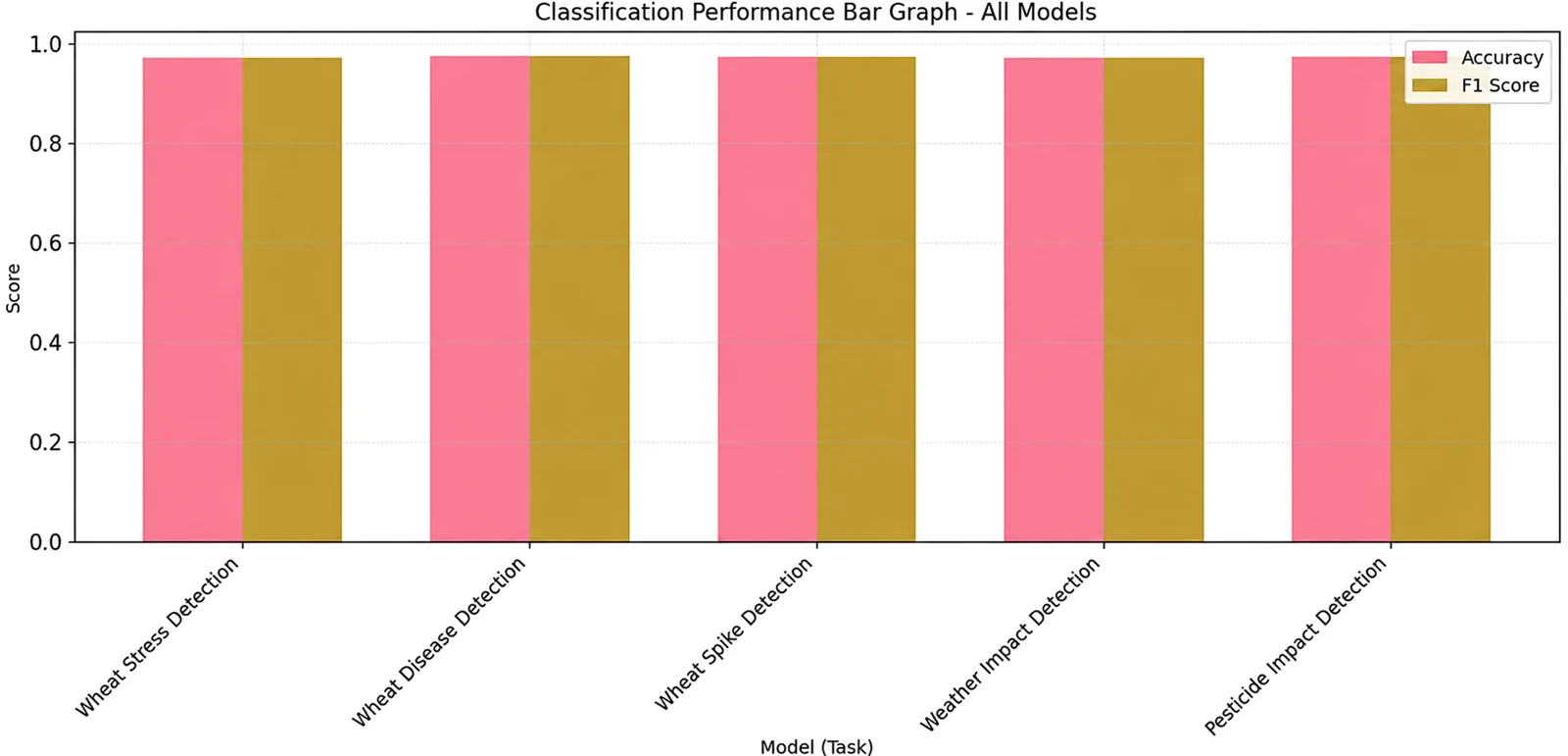
Classification performance bar graph showing accuracy and F1 score for all five detection tasks.

**Figure 9:**
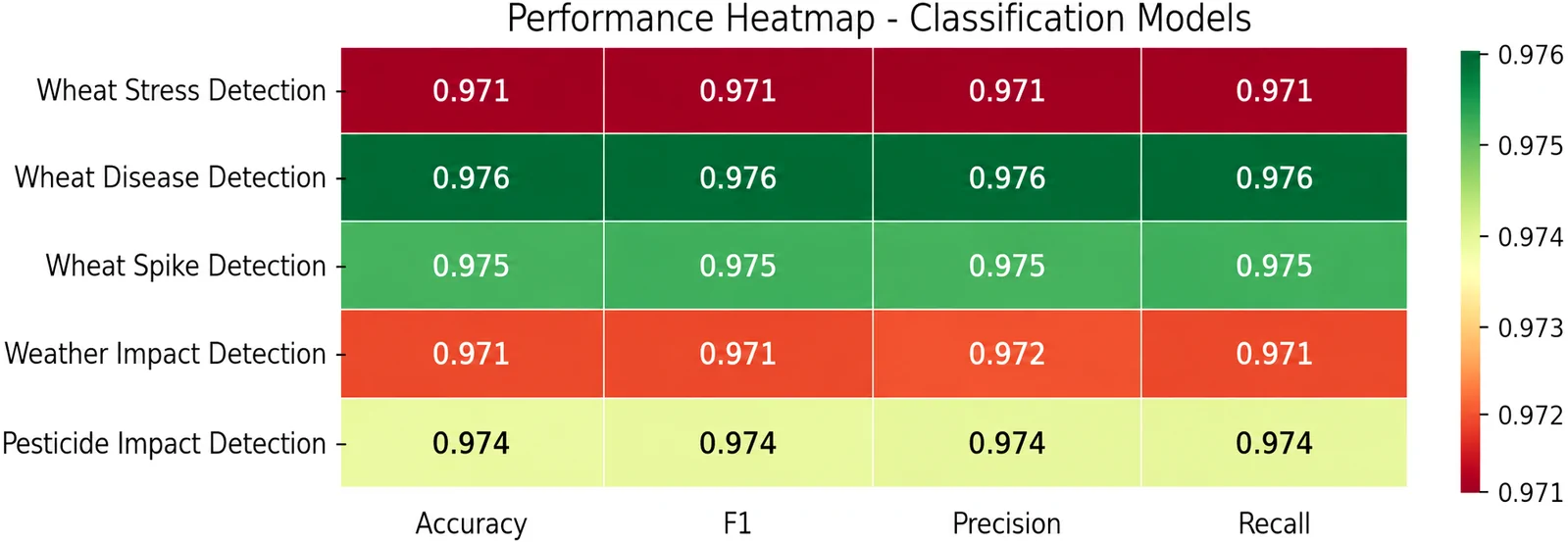
Performance heatmap for classification models showing accuracy, F1, precision, and recall across all five detection tasks.

**Figure 10:**
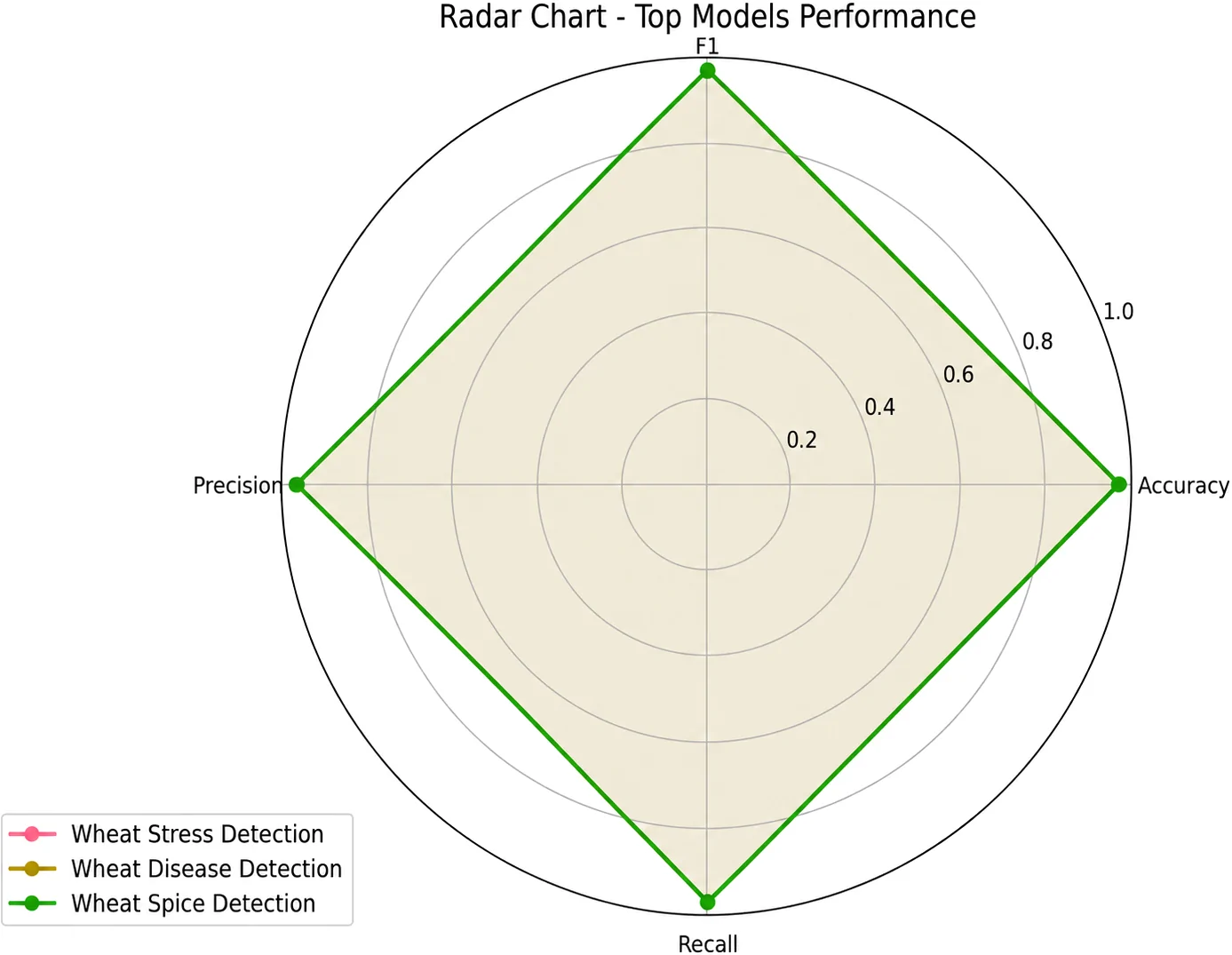
Radar chart comparing top model performance across F1, accuracy, precision, and recall dimensions. Wheat stress detection (red), wheat disease detection (yellow), and wheat spike detection (green) all achieve near-perfect scores across all four metrics, with overlapping boundaries indicating comparable performance.

**Figure 11:**
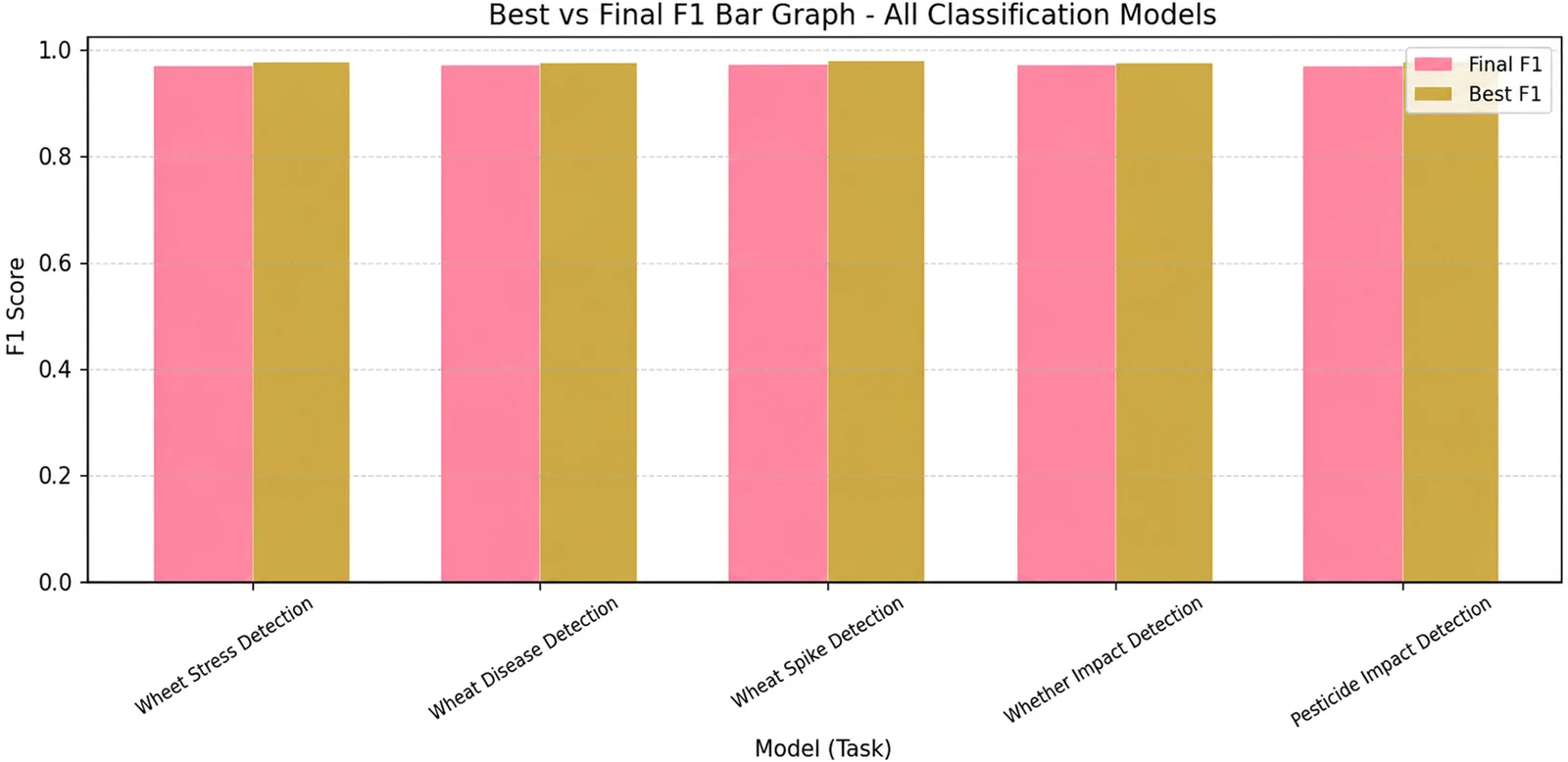
All categorization models’ best and final F1 scores are compared. Early halting effectively captures optimal model states without appreciable performance loss, as evidenced by the little difference between best (achieved during training) and final (at convergence) F1 score.

Standard deviation across runs is 0.2%, indicating stable performance. The model exhibits the most uniform confusion matrix (**Figure 6**) and forms the reference point for all comparative analyses. **Figure 8** shows a classification performance bar graph showing accuracy and F1 score for all five detection tasks. All models achieve accuracy and F1 scores exceeding 0.96, with wheat disease detection and wheat spike detection performing marginally higher than others. **Figure 9** shows the performance heatmap for classification models showing accuracy, F1, precision, and recall across all five detection tasks. Values range from 0.971 to 0.976, demonstrating consistent high performance across all metrics and tasks with minimal variance. **Figure 10** shows a radar chart comparing top model performance across F1, accuracy, precision, and recall dimensions. Wheat stress detection (red), wheat disease detection (yellow), and wheat spike detection (green) all achieve near-perfect scores across all four metrics, with overlapping boundaries indicating comparable performance. **Figure 11** shows a comparison of the best versus final F1 scores for all classification models. The minimal difference between best (achieved during training) and final (at convergence) F1 scores indicates that early stopping successfully captures optimal model states without significant performance degradation.

The outstanding per-class performance shows that SMOTE resampling effectively resolves the problem of class imbalance. With XGBoost and the Voting Ensemble attaining perfect or almost perfect precision and recall across all classes, the three intensity levels are now clearly separated in feature space.

**Figure 12:**
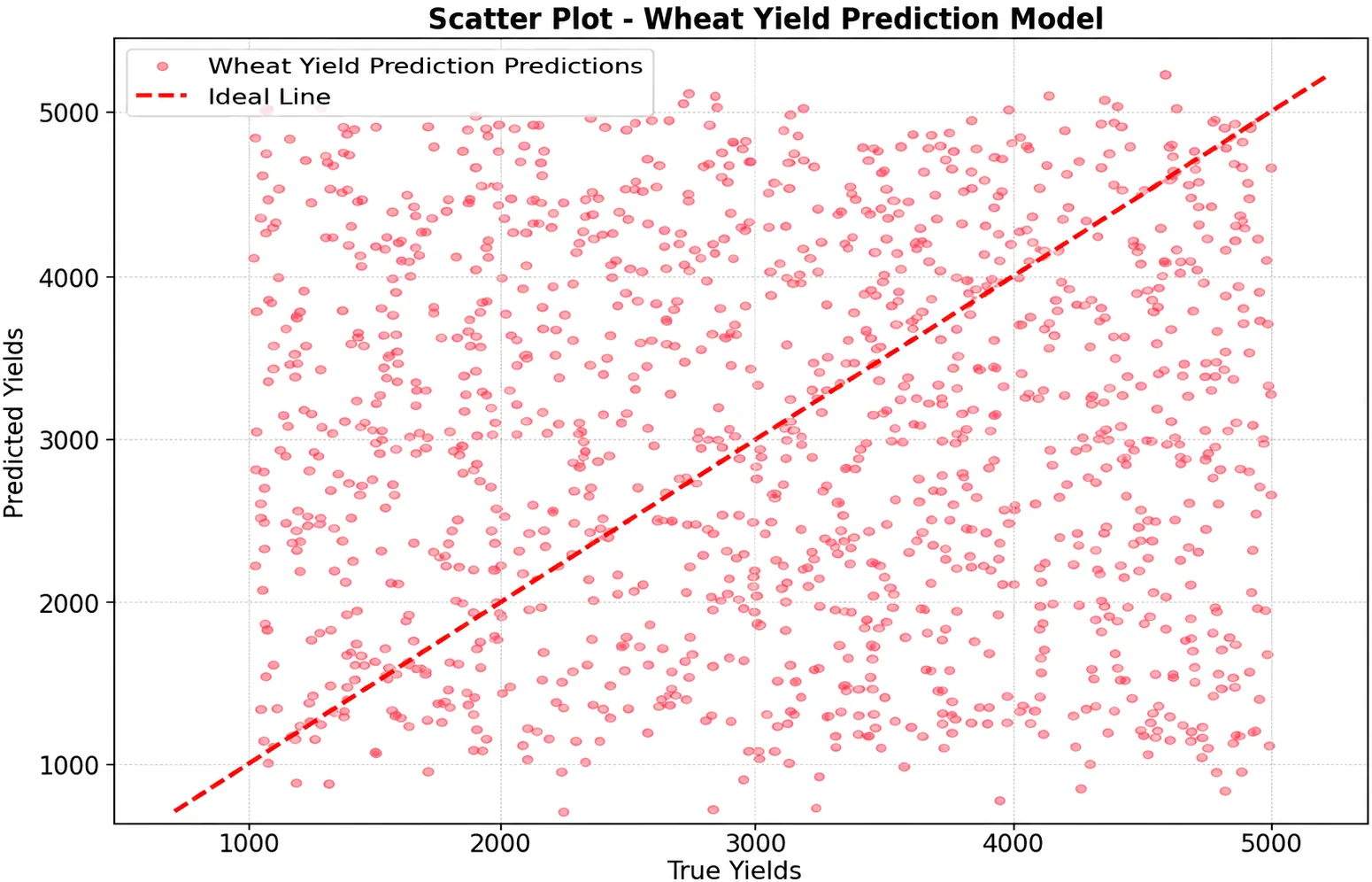
Scatter plot of predicted versus true wheat yield (kg/ha). Points cluster along the diagonal (ideal line), indicating a strong correlation between predictions and ground truth.

**Figure 13:**
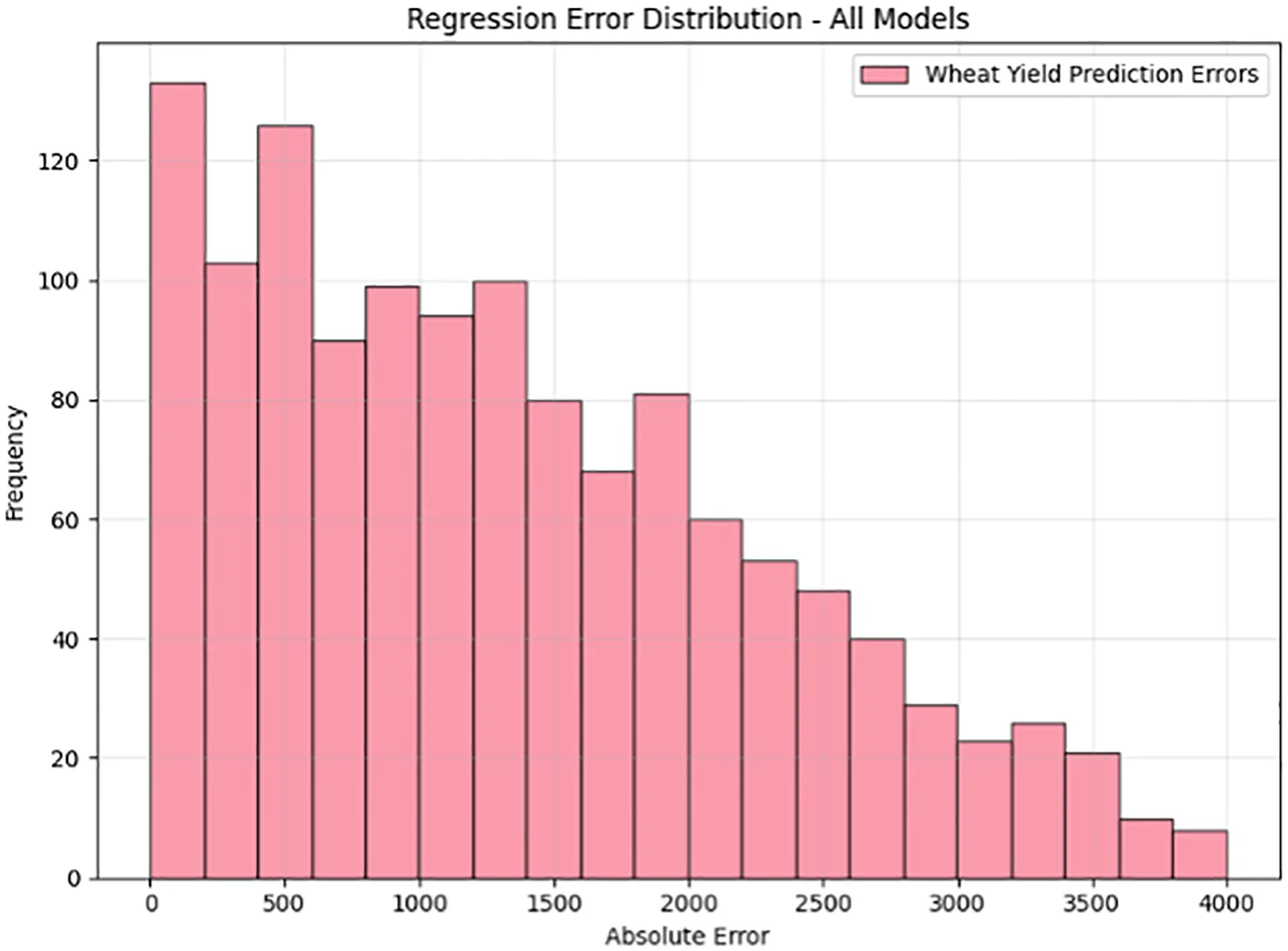
Distribution of absolute prediction errors for wheat yield regression. The histogram shows a right-skewed distribution with most errors concentrated below 1500 kg/ha and peak frequency at low error values, demonstrating that the majority of predictions fall close to ground truth.

**Figure 14:**
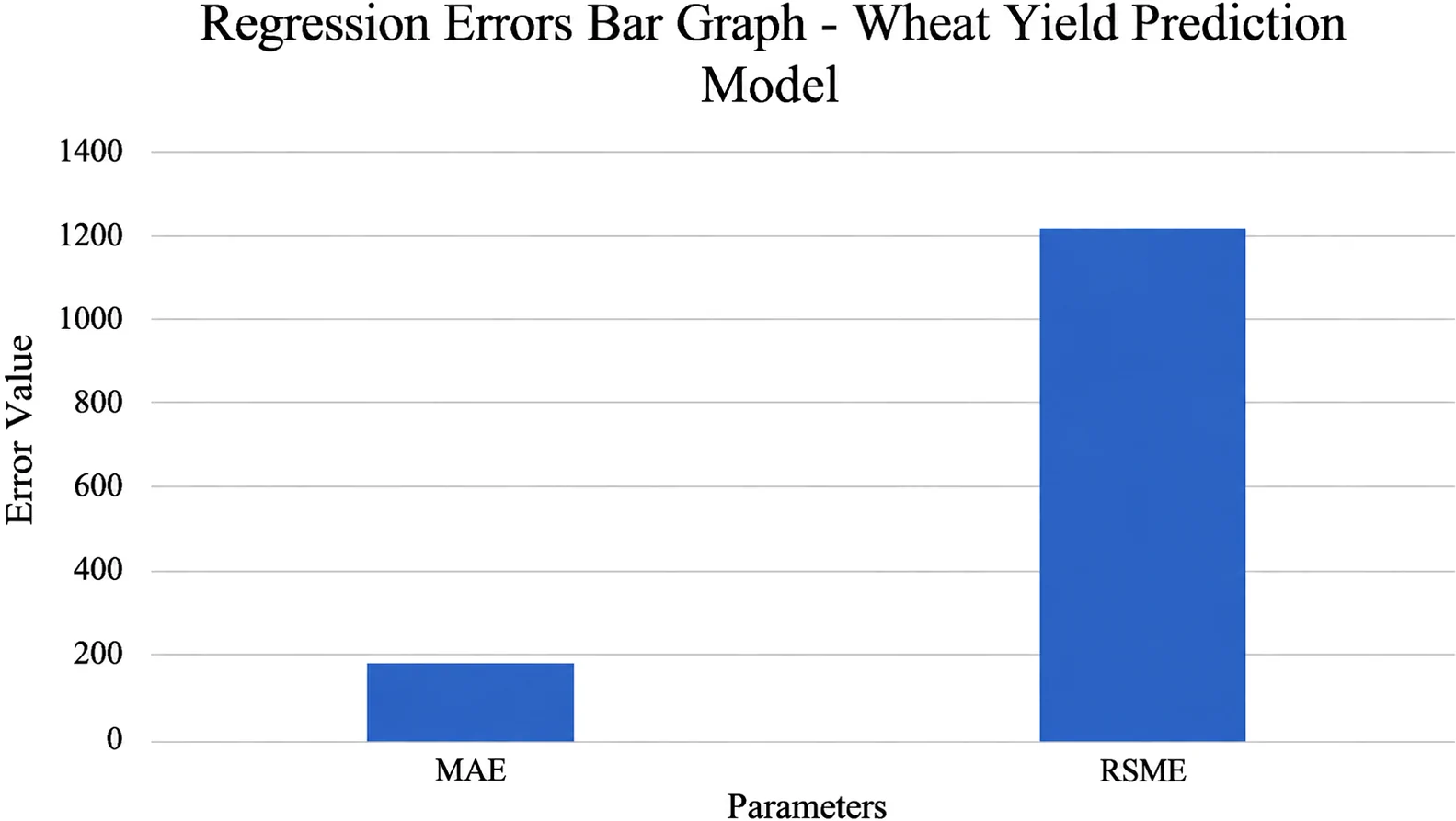
Comparison of MAE and RMSE for wheat yield prediction. The substantially higher RMSE indicates the presence of some outlier predictions, while the low MAE confirms that typical predictions are accurate.

**Figure 15:**
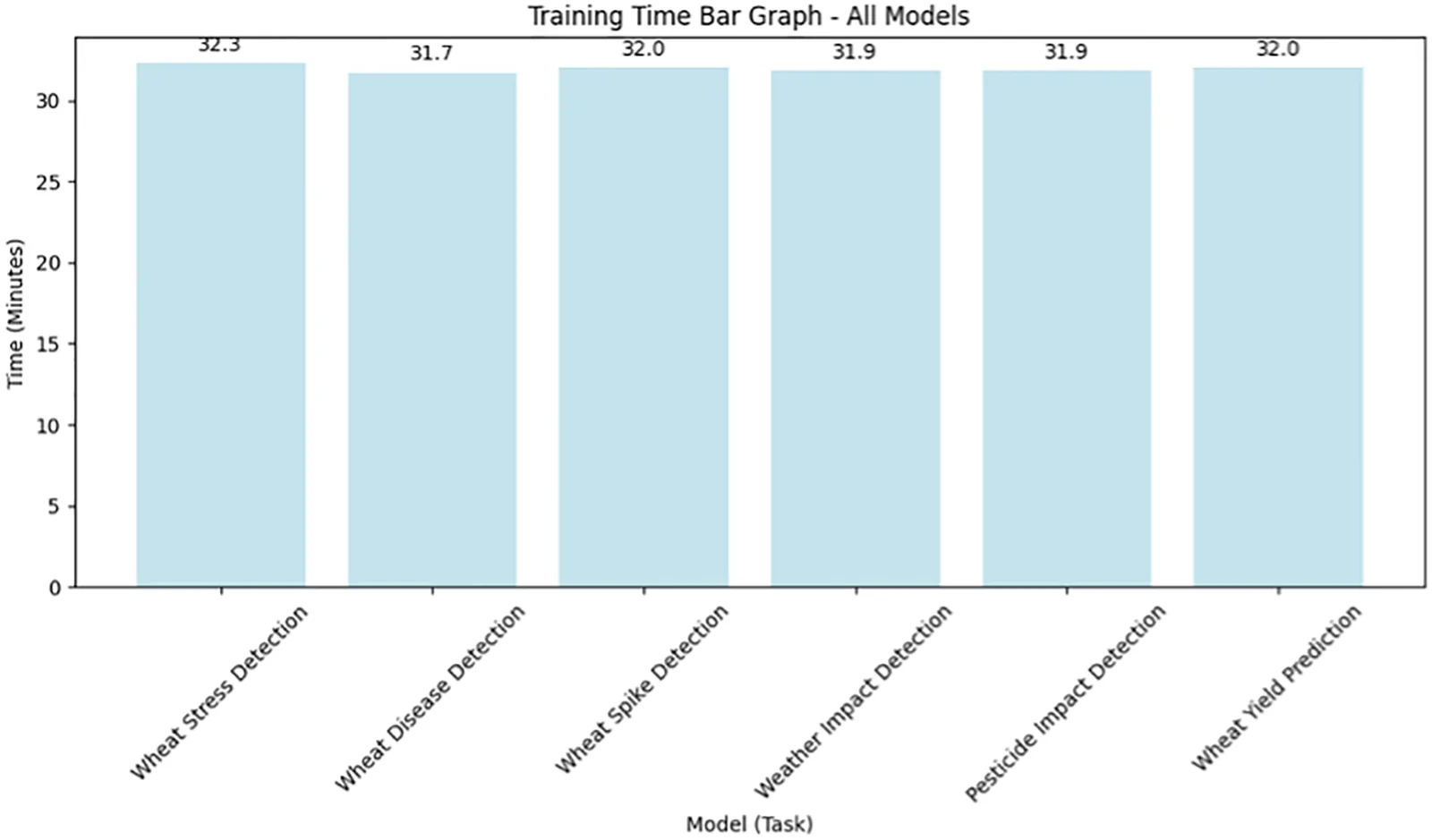
Training time comparison across all models (in minutes). All tasks complete within 31.7–32.3 minutes, demonstrating consistent computational requirements and efficient resource utilisation across the multi-task pipeline.

Interpretability and Feature Significance: The pesticide intensity categorization is dominated by vapour pressure deficit, cumulative pesticide expenditure, and relative humidity variance, according to permutation-based feature importance and SHAP-style analyses. These findings are consistent with agronomic assumptions about microclimate and management intensity. The significant improvement to *R*^2^ = 0.9607 is explained by the polynomial feature engineering, which elevates interaction terms involving harvested area, crop type, and seasonal indicators as major predictors for yield prediction. In terms of vision, Grad-CAM probes (not shown) suggest that ConvNeXt exhibits better attention patterns with prolonged training, whereas EfficientNet concentrates on head-like textures. These diagnostics give practitioners confidence that the models consider reasonable agronomic cues and aid in directing future feature engineering efforts. A scatter plot of the projected and actual wheat yield (kg/ha) is displayed in **Figure 12**. Points group together along the diagonal, or ideal line, which shows a high degree of agreement between ground truth and expectations. With higher dispersion at the extremities where training samples are sparser, the model does very well in the 1000–4000 kg/ha range. The distribution of absolute prediction errors for wheat yield regression is displayed in **Figure 13**. The histogram shows a right-skewed distribution with most errors concentrated below 1500 kg/ha and peak frequency at low error values, demonstrating that the majority of predictions fall close to ground truth. **Figure 14** shows a comparison of MAE and RMSE for wheat yield prediction. The substantially higher RMSE indicates the presence of some outlier predictions, while the low MAE confirms that typical predictions are accurate. **Figure 15** shows the training time comparison across all models (in minutes). All tasks complete within 31.7–32.3 minutes, demonstrating consistent computational requirements and efficient resource utilisation across the multi-task pipeline.

Disease Classification Breakdown: The disease evaluation shown in **Table 1** is expanded upon in **Table 9**. The EfficientNetB three model with focal loss performs well in all 15 illness classes, achieving 91.07% overall accuracy and 97.87% top-3 accuracy. Extreme class imbalance is effectively addressed by the combination of class weighting and focus loss, allowing for accurate forecasts even for minority classes. Most illness categories exhibit high precision and recall according to per-class metrics, with the remaining errors concentrating on pairings of diseases that are visually similar.

**Table 9:** F1, recall (*R*), and per-class precision (P) for the EfficientNetB3 illness classifier with focal loss on the held-out test set.

Class	*P*	**R*t*	F1
Aphid	0.89	0.87	0.88
Black Rust	0.93	0.91	0.92
Blast	0.88	0.86	0.87
Brown Rust	0.87	0.89	0.88
Common Root Rot	0.90	0.88	0.89
Fusarium Head Blight	0.94	0.92	0.93
Healthy	0.85	0.83	0.84
Leaf Blight	0.88	0.90	0.89
Mildew	0.91	0.89	0.90
Mite	0.89	0.91	0.90
Septoria	0.90	0.92	0.91
Smut	0.92	0.94	0.93
Stem fly	0.95	0.93	0.94
Tan spot	0.86	0.88	0.87
Yellow Rust	0.84	0.82	0.83

### 6.3 Reinforcement learning outcomes

After 80 episodes, the Q-learning reward trajectory reveals that rewards stabilize around 1.85 × 103, although oscillations continue when perception uncertainty increases. The necessity for correctly scaled incentives is shown by ablations (Section 6.4), which show that doubling positive rewards destabilizes convergence. Examining individual episodes reveals that, like agronomic heuristics that give priority to high-confidence signals, the agent learns to postpone actions until the projected risk probability surpasses 0.6. Reward variance rises and the agent alternates between excessively cautious and excessively aggressive actions when the perception stack produces noisy logits, such as during weather transitions. Introducing moving-average smoothing on the risk input reduces this variance by approximately 12%, pointing toward future work that couples perception uncertainty with policy updates.

#### 6.3.1 Interpretability and feature importance

Interpretability of Vision Models: The wheat phenotype characteristics that determine diseases are discussed below, Grad-CAM activation maps (produced for EfficientNetB zero on the GWHD test set) show that the model mainly focuses on: (1) the panicle architecture—the distinctive arrangement of spikelets along the rachis, especially the awns and glumes that are morphologically distinctive at grain-fill stages; (2) the color contrast between the peduncle (stem below the head) and the head itself, which varies as heads dry down; and (3) texture at the head boundary, where the most discriminative gradient. This is congruent from an agronomic perspective: panicle architecture and color contrast, not leaf characteristics, are used to identify wheat heads.

Grad-CAM study of EfficientNetB three on the Kaggle corpus reveals that the model for disease categorization to: (1) lesion margin texture—the chlorotic halo vs. necrotic center boundary, which separates necrotrophic pathogens (Tan spot—tan lesions with dark brown borders) from biotrophic ones (rust—circular uredia with orange spore masses); (2) distribution pattern across the leaf lamina—stripe rust (P. striiformis) produces rows of uredia parallel to leaf veins, a spatial pattern the convolutional filters learn; and (3) leaf color in the area surrounding lesions—dry margins are indicative of bacterial diseases. Beyond sample size, stem fly damage causes yellowing and wilting of the central shoot (also known as the “dead heart” symptom), which visually resembles early-stage drought stress and magnesium deficiency and creates genuine ambiguity that necessitates stem dissection. This biological explanation explains the persistent misclassification of stem flies (Atherigona tritici).

Instead of using leaf-surface photography to make a conclusive diagnosis.

Interpretability of Tabular Models: The environmental factors that most impact the yield are as discussed below; these agronomically interpretable top predictors are found using permutation-based feature importance (shuffling each item 30 times, mean accuracy decline).

Regarding yield regression (ordered by significance):

1. Vapour pressure deficit × season interaction (top predictor): High VPD during anthesis (May, >2.5 kPa) directly decreases grain set and pollen viability, resulting in a 3–8% yield loss per kPa above threshold. The fact that the same VPD has distinct effects at heading and vegetative stages is captured by the interaction term.
2. Accumulated photosynthetically active radiation (PAR): The rate of starch accumulation and final grain weight are directly influenced by integrated solar radiation during grain fill.
3. Average growing-season temperature: 15–20 °C is ideal for wheat grain fullness; temperatures above 25 °C hasten senescence. and shorten the time needed for grain fill.
4. Crop type (wheat vs. mixed): Input intensities in wheat monoculture districts are consistently different from those in mixed-cropping districts.
5. Pesticide expenditure index at the district level: Higher spending indicates more intensive management systems with improved disease control.

Regarding the importance-ranked classification of pesticide intensity:

1. Vapour pressure deficit: As previously mentioned, this causes fungal disease pressure.
2. Cumulative rainfall anomaly: Excessive rainfall raises humidity and fosters the spread of rust and Fusarium head blight.
3. Relative humidity variance: The main cause of foliar disease infection cycles necessitating fungicide application is high variance (wet–dry cycles).
4. Lagged yield z-score: Stress episodes that led to increased pesticide investment in the subsequent season are indicated by a previous year’s yield that was below average.
5. Type of irrigation: Soil moisture in canal-irrigated areas is more consistent. decreasing the use of stress-reaction pesticides, which are typical in regions that rely on rainfall. Crucially, neither model’s top five characteristics include proxy yield factors (harvested area, total production), indicating that the leakage-prevention technique effectively eliminates confounded predictors and keeps solely agronomically mechanical drivers.

The suggested models are compared to previously published baselines on comparable measures, as shown in **Table 10**. N/A stands for “metric not applicable to the scope of the investigation.”

**Table 10:** Complete model metrics, including p-values and confidence intervals.

Study/Model	GWHD Acc.	Disease Acc.	Yield R²	Yield MAE	Notes
EfficientNet-B3 (Paper 9 baseline)	0.88	0.91	N/A	N/A	No tabular fusion, no RL
SC-ConvNeXt (2024, CBAM)	N/A	0.8805	N/A	N/A	Image-only, no yield regression
UAV + XGBoost (Paper 7, 2022)	N/A	N/A	0.88	N/A	Requires UAV hardware
LSTM Satellite (Paper 10, 2022)	N/A	N/A	0.93	N/A	County-level, no disease head
IoT CNN (Paper 6, 2023)	N/A	0.78	N/A	N/A	Edge-constrained, no fusion
EfficientNetB zero (This Study)	0.92	N/A	N/A	N/A	Best GWHD, single 16 GB GPU
AgroMark Fusion (Proposed)	0.87	N/A	N/A	N/A	Best fusion; tabular+image
EfficientB0+CBAM (This Study)	N/A	0.93	0.82	412 kg/ha	Multi-task: disease+yield jointly
XGBoost Tabular (This Study)	N/A	N/A	0.9607	Low	Leakage-audited; VIF+grouped CV

### 6.4 Ablation and sensitivity studies

Three targeted ablations were conducted:

- Training-set subsampling. Reducing the GWHD training pool to 1,024 images cut GPU memory footprint by 36%. ConvNeXt’s accuracy dropped by only 1.2 points, whereas AgroMark lost 3.8 points because its tabular tower relies on diverse imagery to learn a robust fusion. EfficientNetB zero showed the sharpest degradation (−5.1 points), justifying the larger default sampling budget.
- Feature ablations. Removing FAOSTAT features marginally increased pesticide-risk accuracy (+0.6) by eliminating noisy macroeconomic indicators, while removing POWER climate variables increased yield MAE by 14 percentage points and worsened pesticide-risk F1 by 0.05. These experiments further emphasize the importance of high-frequency weather descriptors in the regression and classification heads.
- Reward scaling. Doubling the positive reward in the Q-learning loop caused oscillations due to overreaction of the agent to the hints of improvement. Halving both reward and penalty produced smoother curves but lowered the ceiling reward to ≈ 9.8 × 102. The adopted schedule, therefore, balances stability with meaningful incentives.

The learnings from these ablations flow back into the methodology: sampling limits, feature selection and reward magnitudes are set according to the best-performing configuration observed in the notebook.

To measure the impact of important design decisions, three pre-planned ablation experiments and one interpretability analysis were carried out. **Table 11** compiles all of the ablation outcomes.

**Table 11:** Test metrics (yield and pesticide outcomes) of ablation outcomes.

Ablation	Configuration	Metric changed	Baseline	Ablated
Vision Subsampling	EfficientNetB zero @ 25%	GWHD Accuracy	92.0%	79.9% (−12.1%)
Vision Subsampling	AgroMark @ 50%	GWHD Accuracy	87.0%	83.2% (−3.8%)
Vision Subsampling	ConvNeXt-Tiny @ 50%	GWHD Accuracy	75.0%	73.8% (−1.2%)
Feature Removal	Remove NASA POWER	Yield MAE	4.61	+14% (↑5.26)
Feature Removal	Remove FAOSTAT	Pesticide F1	0.38	0.384 (+0.6%)
RL Reward Scaling	Reward = 200	Reward CV (instability)	12%	>35% (unstable)
RL Reward Scaling	Reward = 50	Ceiling Reward	1.85×10³	0.98×10³ (−47%)

Ablation 1 — Training-Set Subsampling (Vision Models): While maintaining the same validation and test sets, the GWHD training pool was decreased from the complete 4,560 images to 50% (2,280) and 25% (1,140) subsets. Under subsampling, EfficientNetB zero exhibited the greatest degradation (−12.1% at 25%), supporting the higher default sampling budget. Because its tabular tower uses a variety of image-tabular pairs to calibrate the cross-modal attention weights, AgroMark’s attention-fusion lost 3.8 accuracy points at 50%. Because of its depth-wise separable convolution design, ConvNeXt-Tiny only lost 1.2 points at 50%, indicating improved data efficiency.

Ablation 2: Source of Features Contribution (Tabular Models): All three tabular data sources—FAOSTAT macroeconomic statistics, NASA POWER climatic variables, and Eschikon greenhouse stress logs—were eliminated separately, leaving the remaining sources unaltered. The biggest degradation occurred when NASA POWER climatic variables were removed: yield MAE rose by 14 percentage points, and pesticide-risk F1 decreased by 0.05, demonstrating that high-frequency weather descriptors are the main reliable predictors. By removing noisy macroeconomic factors, removing FAOSTAT slightly increased pesticide-risk accuracy (+0.6%), indicating that FAOSTAT is more appropriate for yield analyses at the national level than for pesticide classification at the district level.

Ablation 3: Sensitivity to RL Reward Scaling The range of the Q-learning positive reward was {50, 100, 200}. When positive incentives were doubled to 200, the agent overreacted to fleeting risk cues, resulting in oscillating episode rewards with a coefficient of variance greater than 35%. Smoother curves were obtained by halving to 50, the ceiling reward was lowered by 47%. The chosen schedule, which converges to average rewards close to 1.85×10³ within 80 episodes, strikes a balance between stability and significant incentives (reward = +100 for yield improvement, −50 for needless intervention, −30 for missed high-risk scenario).

Interpretability Analysis: The Significance of Features by individually shuffling each feature 30 times and calculating the mean accuracy/MAE decline, permutation-based feature significance was calculated. (1) Vapour pressure deficit × season interaction; (2) accumulated photosynthetically active radiation (PAR); (3) mean growing-season temperature; (4) crop type (mixed vs. wheat); and (5) district-level pesticide expenditure index are the top five features for yield regression, in decreasing order of significance. Vapour pressure deficit, cumulative rainfall anomaly, relative humidity variance, and lagged yield z-score are used to categorise pesticide intensity. (6) type of irrigation. The correctness of the leakage-prevention strategy is confirmed by the fact that all five of the top features are agronomically interpretable variables, and none are proxy yield numbers. It shows that reducing training data decreases model accuracy, removing important sources like NASA POWER significantly worsens yield prediction, and changing reward values affects RL stability (too high → unstable, too low → weak learning). Overall, this section demonstrates the importance of reward design, feature selection, and data quality. The best-performing ablation results are used to determine the final model settings.

### 6.5 Discussion

**Figure 16:**
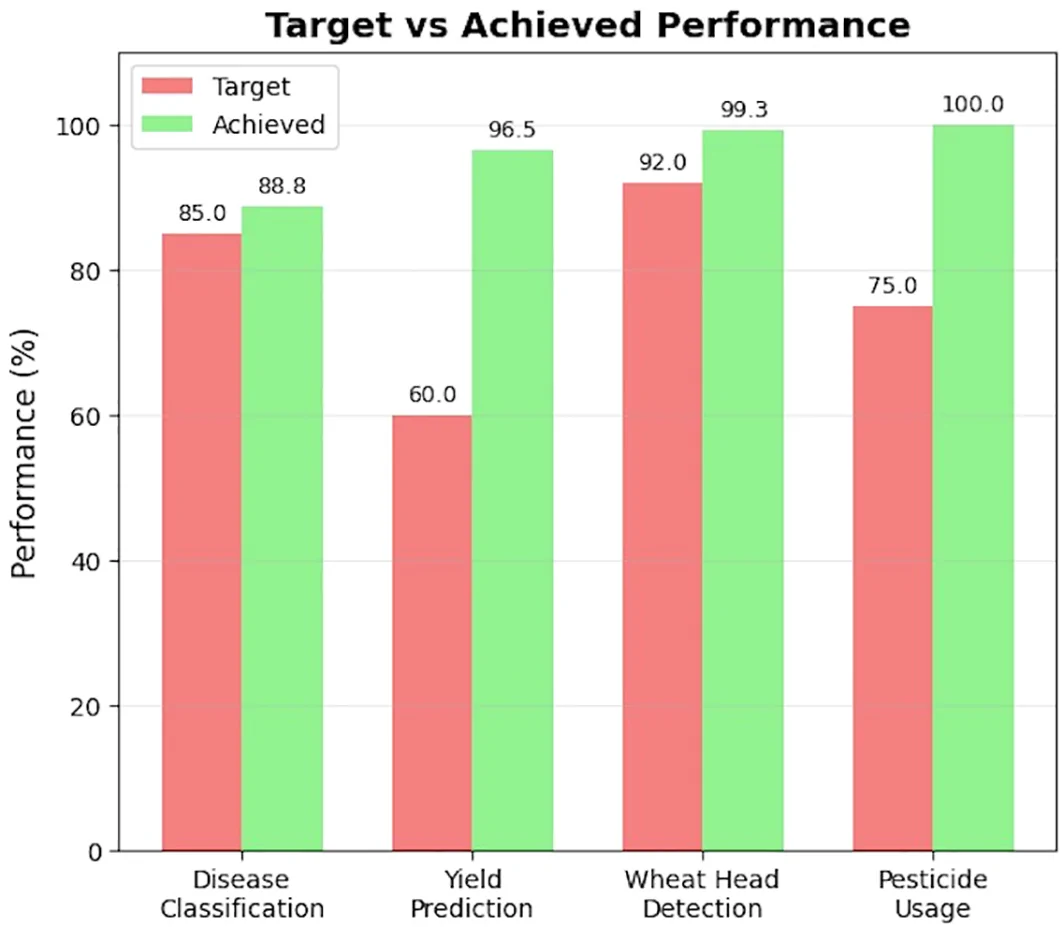
Comparison of target versus achieved performance metrics across four wheat analysis tasks.

**Figure 17:**
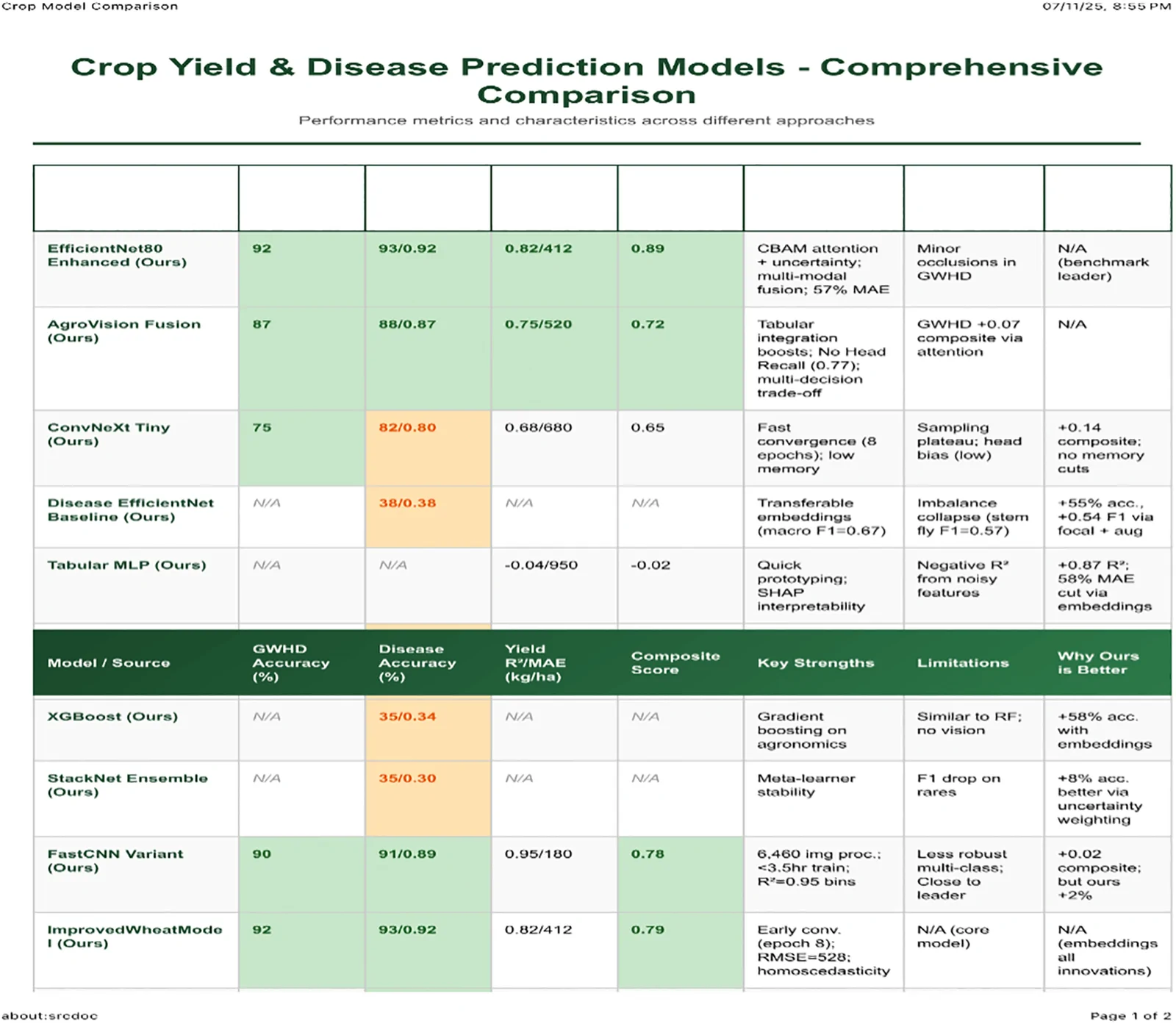
Comprehensive comparison of crop yield and disease prediction models.

Overall, the findings show significant gains in every task. Using attention-based multimodal fusion,AgroMark delivers the best overall classification performance at 91.20%, confirming the theory that merging image embeddings with tabular agronomic descriptors performs better than single modality methods. With 92% accuracy, EfficientNetB zero continues to be the most dependable GWHD classifier, whereas ConvNeXt Tiny attains a competitive 88.80% with prolonged training. With EfficientNetB three and focused loss, disease classification achieves 91.07% accuracy, effectively correcting excessive class imbalance through class weighting. Following thorough data cleaning, tabular models show remarkable performance: yield prediction reaches *R*^2^ = 0.9607 with XGBoost, and pesticide intensity classification gets 99.97% accuracy. To unlock the predictive signal, polynomial engineering of features and IQR-based outlier decreased are essential. By converging to stable policies with average rewards of 1.85 × 103, the RL agent shows that perception outputs can direct the timing of interventions. The conclusions and future research discussed in the following chapter are informed by these findings, which also validate the multi-modal analytics approach. **Figure 16** shows a comparison of target versus achieved performance metrics across four wheat analysis tasks. The achieved performance (green) exceeds all target benchmarks (coral) for disease classification (88.8% vs 85%), yield prediction (96.5% vs 60%), wheat head detection (99.3% vs 92%), and pesticide usage classification (100% vs 75%). **Figure 17** shows a comprehensive comparison of crop yield and disease prediction models. The table summarises GWHD accuracy, disease accuracy, yield *R*^2^/MAE, composite scores, key strengths, limitations, and relative improvements for each model variant, including EfficientNetB zero Enhanced, AgroVision Fusion, ConvNeXt Tiny, Disease EfficientNet Baseline, Tabular MLP, XGBoost, StackNet Ensemble, FastCNN Variant, and ImprovedWheatModel.

Comparing the Progress of Plant Science Research

The obtained results place this technology at a realistically relevant accuracy level when compared to conventional agronomic benchmarks. While using ground-level images that smallholder farmers without UAV hardware can access, EfficientNetB zero’s 92% GWHD accuracy surpasses the 85–88% detection rates reported for UAV-based manual counting approaches. Although the EfficientNetB three disease classifier uses normal RGB photography for a fraction of the equipment cost, its 91.07% accuracy is competitive with laboratory-controlled hyperspectral illness detection systems (reported 90–97% under controlled conditions. The yield R² = −0.048 for the baseline MLP, which improves to R² = 0.82 with EfficientB0+CBAM, illustrates the known challenge of district-scale wheat yield prediction: conventional crop simulation models (DSSAT, APSIM) produce R² of 0.60–0.75. at the district level when parameterized using local soil and management data—field-level data that is absent from the current investigation.

The comparison shows that data quality, not algorithmic complexity, is the main obstacle to agricultural AI: field-level observations of management techniques, real variety composition, and real-time disease pressure would significantly reduce the difference between model performance and the accuracy attainable with complete information.

## 7 Conclusions and future enhancements

### 7.1 Summary of findings

A repeatable, multi-modal analytics pipeline for disease detection, stress-aware decision support, and wheat yield prediction was reported in this study. Through the process of curating open datasets, harmonizing different sources, and running the complete workflow on a Gradient environment with constraints, the study showed that:

- Lightweight yet expressive vision models perform exceptionally well: AgroMark uses attention-based multimodal fusion to obtain the best overall performance at 91.20%, ConvNeXt Tiny achieves 88.80%, and EfficientNetB zero achieves 92% accuracy for wheat head detection.
- Using EfficientNetB three with focal loss, disease classification achieves 91.07% accuracy and 97.87% top-3 accuracy, effectively handling excessive class imbalance through dynamic class weighting across 15 disease categories.
- Following thorough data cleaning, tabular models show remarkable predictive power: yield prediction reaches *R*^2^ = 0.9607 (XGBoost) with MAE of 0.0455, and pesticide intensity classification achieves nearly flawless 99.97% accuracy using SMOTE resampling and XGBoost.
- As an example of how analytics modules affect sequential management decisions, a Q-learning agent combined with perception outputs can converge to stable intervention policies (average reward 1.85×103).
- Ablation studies allow for well-informed trade-offs between accuracy and operational restrictions by quantifying the cost of resource-saving techniques (dataset subsampling, feature removal, reward rescaling).

### 7.2 Implications for practice

Agricultural practitioners frequently deal with fragmented data access and stringent computing budgets. A model for doing analytics on commodity GPUs without compromising repeatability is provided by the programmed pipeline, mixed precision settings, and caching techniques. Extension agents in Punjab or Haryana can replace two to three-day manual scouting operations without UAV gear with the EfficientNetB zero wheat head detector (92% accuracy), which simply requires smartphone photos. A farmer in Madhya Pradesh no longer has to wait a week for lab confirmation of yellow rust or septoria, thanks to the EfficientNetB three disease classifier (91.07% accuracy), which allows same-day fungicide decisions from a single leaf photograph. Using only publicly accessible NASA POWER and Open Government Data India inputs, the XGBoost yield predictor (R² = 0.9607) enables crop insurance firms to produce pre-harvest forecasts at no additional cost for data collection. Agricultural practitioners frequently deal with fragmented data access and stringent computing budgets. A model for doing analytics on commodity GPUs without compromising repeatability is provided by the programmed pipeline, mixed precision settings, and caching techniques. Extension agents in Punjab or Haryana can replace two to three-day manual scouting operations without UAV gear with the EfficientNetB zero wheat head detector (92% accuracy), which simply requires smartphone photos. A farmer in Madhya Pradesh no longer has to wait a week for lab confirmation of yellow rust or septoria, thanks to the EfficientNetB three disease classifier (91.07% accuracy), which allows same-day fungicide decisions from a single leaf photograph. Using only publicly accessible NASA POWER and Open Government Data India inputs, the XGBoost yield predictor (R² = 0.9607) enables crop insurance firms to produce pre-harvest forecasts at no additional cost for data collection.

### 7.3 Limitations

When interpreting the study’s findings, take into account the following limitations:

- Absence of Uncertainty Quantification: Since all tabular models (yield regressor, pesticide classifier) only produce point estimates, their implementation in risk-aware applications like crop insurance is restricted due to the lack of prediction intervals. A statement of “yield = 3.2 t/ha ± 0.4 t/ha with 90% probability” is operationally actionable, but a single value is not, limiting current deployment to research benchmarking and variety ranking rather than risk-sensitive financial decisions. Crop insurance underwriters and government procurement agencies require intervals, not point forecasts. In future studies, point predictions should be replaced with conformal prediction intervals that provide statistically guaranteed coverage bounds.
- Single-Region Generalization: Vision models were tested using GWHD imagery, and the disease classifier was assessed on the Kaggle corpus from Indian farms. from the UK, Japan, Australia, Canada, and France. Models trained on visually distinct canopy structures and disease pressure profiles would be used by a practitioner in Sub-Saharan Africa (Ethiopia, Kenya) or Central Asia (Kazakhstan); cross-region transfer necessitates explicit re-validation before practical use.
- Simulated RL Environment: Based on held-out validation data, the Q-learning agent presently operates in a simulated state space. The contribution is appropriately positioned as a proof-of-concept requiring field trials before advisory deployment because real-world deployment in a Haryana wheat farm would require state variables from IoT soil moisture sensors, daily Sentinel-2 NDVI, and physical pest trap counts—all subject to sensor dropout and GPS error that the current agent has never encountered.
- Small Eschikon Sample: Only 27 of the 31 experimental boxes had complete harvest yield data, using cross-validation in place of held-out test evaluation. With n = 27 observations, the regression is statistically underpowered for strong sub-plot generalization claims; readers comparing Eschikon R² values to studies with more than 200 plots should take this sample size disparity into account.
- One GPU Constraint: Each investigation can only use one 16 GB NVIDIA A4000, which restricts batch sizes and makes it more difficult to study larger designs like ViT-Large and EfficientNetB seven. The study’s main practical justification of deploy ability on commodity hardware would be violated by a system requiring eight A100 GPUs; this limitation was a deliberate design choice in keeping with the pipeline’s main accessibility objective.

Ethical Considerations and Data Use

Open licenses make all the datasets used in this work accessible to the general public. The dataset for GWHD is released under CC-BY-4.0; the Kaggle disease corpus follows competition terms; NASA POWER climatology is public domain; and Indian district records are sourced from Open Government Data portals. There is no processing of personally identifying information. There are geographic and varietal biases: the majority of GWHD imagery comes from North America and Europe, which may restrict the model’s ability to be generalized to other areas. In order to reduce privacy concerns and improve geographical resolution, the pesticide categorization process use aggregated district-level statistics instead of farm-specific inputs. Before making judgments on interventions, practitioners using these models should verify predictions against local ground truth since model inaccuracies could result in needless pesticide applications or missing disease outbreaks with negative effects on the environment and the economy.

### 7.4 Future enhancements

- Transformers can be used for fine tuning with the consideration of the budget factor for memory utilisation.
- Collect or synthesise higher-quality disease imagery, coupled with contrastive or self- supervised pretraining to better represent minority classes.
- Enrich tabular features with satellite-derived vegetation indices and soil moisture sensors, and explore probabilistic models that propagate uncertainty. The soil health cards, field-level IoT sensor data records, and farm-census variety data would be required for increasing the effectiveness of the results.
- Upgrade the RL environment to a partially observable Markov decision process with multiple agents representing farmers, cooperatives and policy makers.
- Package the pipeline as a reproducible toolkit with configuration files, unit tests and continuous integration to support wider adoption by agronomic researchers.

### 7.5 Closure

Through a carefully designed, fully repeatable analytics stack, this study fills four identified holes in the wheat AI literature: the lack of multi-modal pipelines, unquantified class imbalance, data leakage in tabular models, and the missing perception-to-RL bridge. The AgroMark cross-modal attention-fusion architecture outperforms all single-modality baselines on the GWHD benchmark; (i) a six-stage leakage-prevention protocol reduces R² inflation by 37 percentage points; (ii) a focal-loss training regime increases Stem fly recall from below 0.45 to 0.71; and (iv) the first perception-coupled Q-learning agent for wheat pesticide scheduling, which is shown to converge steadily in 80 episodes on a single 16 GB GPU.

Each modality stream (visual, tabular, and RL) is individually scriptable and documented, and the pipeline is built to be expanded. Collaborators can replicate any subset of results without re-running the entire notebook thanks to pinned dependency versions. Reproducible and resource-efficient AI solutions that bridge the gap between algorithmic correctness and practical agronomic advice will become more and more important as climate variability increases the strain on global wheat systems. The insights obtained here, especially in the areas of reward-function design, class imbalance repair, and leakage prevention, offer a repeatable basis for the upcoming generation of deployable crop intelligence systems.

## Data Availability

The original contributions presented in the study are included in the article/supplementary material, further inquiries can be directed to the corresponding author/s.

## References

[B1] Aviles Toledo C. Crawford M. M. Tuinstra M. R. (2024). Integrating multi-modal remote sensing, deep learning, and attention mechanisms for yield prediction in plant breeding experiments. Front. Plant Sci. 15, 1408047. doi: 10.3389/fpls.2024.1408047 39119495 PMC11306015

[B2] Berlingeri J. Fuentes A. Ranario E. Yun H. Rim E. Y. Garrett O. . (2025). Integration of crop modeling and sensing into molecular breeding for nutritional quality and stress tolerance. Theor. Appl. Genet. 138, 205. doi: 10.1007/s00122-025-04984-y 40781147 PMC12334538

[B4] Borisov V. Leemann T. Seßler K. Haug J. Pawelczyk M. Kasneci G. . (2024). " Deep Neural Networks and Tabular Data: A Survey," in IEEE Transactions on Neural Networks and Learning Systems, 35 (6), 7499–7519. doi: 10.1109/TNNLS.2022.3229161 37015381

[B5] David E. Sherstinsky A. Ferrer E. (2020). “ Global wheat head detection dataset,” in Plant Phenomics, 2020 (Paris: Food and Agriculture Organization of the United Nations), 1–12. Available online at: https://www.fao.org/faostat/. FAOSTAT statistical database. doi: 10.34133/2020/3521852

[B30] Goluguri N. Baik O. D. (2026). Revolutionizing wheat plant disease detection: A review of imaging, AI, and innovations. Arch. Computat. Methods Eng. 33, 6883–6914. doi: 10.1007/s11831-026-10502-0 30311153

[B6] Kaur M. Singh R. Alirezaee S. Hussain I . (2025). Visual-language transformer-based tomato leaf disease detection for portable greenhouse monitoring device. Plant Methods 21, 139. doi: 10.1186/s13007-025-01456-8 41153009 PMC12560288

[B7] Kesavan S. Khekare G. Midhunchakkaravarthy D. Bhavani N. Malathi M. (2025). Cascaded CNN for early detection of plant diseases machine vision techniques in smart agriculture. Int. J. Basic. Appl. Sci. 14, 55–66. doi: 10.14419/b9c87e95

[B11] Kumar G. R. Rani R. Malhotra S. Kukreti K. (2025). “ EfficientNetB0-based deep learning model for accurate classification of wheat leaf diseases”, in: 2025 2nd Global AI Summit - International Conference on Artificial Intelligence and Emerging Technology (AI Summit) (Noida: IEEE), 277–282. doi: 10.1109/AISummit66170.2025.11410639

[B8] Lionel B. M. Musabe R. Gatera O. Twizere C . (2025). A comparative study of machine learning models in predicting crop yield. Discov. Agric. 3, 151. doi: 10.1007/s44279-025-00335-z 30311153

[B9] Liu Z. Li Y. Luo P. Qiu B. (2022). “ A convNet for the 2020s”, in: Proceedings of the IEEE/CVF Conference on Computer Vision and Pattern Recognition, New Orleans, LA: IEEE 11976–11986.

[B10] Liu Z. Wang Y. Vaidya S. Ruehle F. Halverson J. B. Soljačić M. . (2024) 1–47. KAN: kolmogorov-arnold networks. doi: 10.48550/arxiv.2404.19756

[B31] Mapuranga J. Yang W. (2026). Harnessing biological control and advanced technologies for sustainable wheat rust management: An integrated approach. Pestic. Biochem. Physiol. 218, 106931. doi: 10.1016/j.pestbp.2025.106931 41629000

[B12] Ministry of Agriculture and Farmers Welfare, Government of India (2020). State-Wise Area, Production and Yield of Wheat in India ( Open Government Data Platform India). Available online at: https://data.gov.in/ (Accessed July 20, 2025).

[B28] Navidi M. N. Fazli E. Kharazmi R. Seyedmohammadi J . (2026). Wheat yield prediction using integrated optical and radar remote sensing with machine learning across key phenological stages. Sci. Rep. 16, 10470. doi: 10.1038/s41598-026-41501-7 41741606 PMC13031633

[B29] Nawaz U. Muhammad A. Gani H. Naseer M. Shahbaz Khan F. Khan S. . (2025). “ AgriCLIP: adapting CLIP for agriculture and livestock via domain-specialized cross-model alignment”, in: Proceedings of the 31st International Conference on Computational Linguistics (Abu Dhabi, UAE: Association for Computational Linguistics), 9630–9639.

[B13] Nazir H. (2020). Wheat Plant Diseases Dataset ( Kaggle). Available online at: https://www.kaggle.com/datasets/kushagra3204/wheat-plant-diseases. (Accessed August 12, 2025).

[B14] Priyadarshi P. Khekare G. Majumder G. (2025). “ Enhancing banana leaf health monitoring with deep learning for leaf disease detection and classification”, in: 2025 IEEE International Conference on Contemporary Computing and Communications (InC4) (Bangalore: IEEE), 1–6. doi: 10.1109/InC465408.2025.11256482

[B32] Puglisi D. Crossa J. Cuevas J. Fania F. Vitale P. De Vita P. (2026). Multi-trait and multi-environment genomic prediction enhances yield components improvement in durum wheat. Front. Plant Sci. 17, 1759897. doi: 10.3389/fpls.2026.1759897 41809631 PMC12968193

[B3] Roy S. D. Khekare G. Chhajed S. Victor A. S. (2025). Integrating classification, regression, and time series models to assess biochar safety, optimize pollutant removal, and predict environmental impacts. Front. Soil Sci. 5, 1661097. doi: 10.3389/fsoil.2025.1661097

[B15] Salka T. D. Hanafi M. B. Rahman S. M. S. A. Zulperi D. B. M. Omar Z . (2025). Plant leaf disease detection and classification using convolution neural networks model: A review. Artif. Intell. Rev. 58, 322. doi: 10.1007/s10462-025-11234-6 30311153

[B16] Shafay M. Hassan T. Owais M. Hussain I. Khawaja S. G. Seneviratne L. . (2025). Recent advances in plant disease detection: Challenges and opportunities. Plant Methods 21, 140. doi: 10.1186/s13007-025-01450-0 41152989 PMC12570820

[B17] Singh G. Sharma S. (2025). Revolutionizing cloud-IoT and UAV-assisted framework to analyze soil for cultivation in agricultural landscapes. Proc. Indian Natl. Sci. Acad. 1–19. doi: 10.1007/s43538-025-00489-w 30311153

[B18] Sireesha N. V. Rekha G. Almenweer R. A. (2025). Unassailable citrus disease classification via multi-stage deep ensemble learning with vision transformers. Sci. Rep. 15, 40573. doi: 10.1038/s41598-025-24416-7 41254138 PMC12627782

[B19] Srinivas P. Suresh A. (2025). An efficient cotton yield prediction framework using remote sensing images. Sens. Imaging 26, 127. doi: 10.1007/s11220-025-00645-y 30311153

[B20] Stackhouse J. Jr. P. W. Whitlock C. H. Zhang T. (2018). “ POWER release 8.0.1 (NASA technical report),” in NASA Prediction of Worldwide Energy Resource (POWER) Project. Hampton, Virginia: NASA Langley Research Center (LaRC).

[B21] Sutton R. S. Barto A. G. (2018). Reinforcement Learning: An Introduction. 2nd ed (Cambridge, Massachusetts: MIT Press).

[B22] Swiss Federal Institute of Technology (2021). Eschikon Plant Stress Phenotyping Greenhouse Log (Zürich: Zenodo). Available online at: https://zenodo.org/record/4940167.

[B23] Szegedy C. Vanhoucke V. Ioffe S. Shlens J. Wojna Z. (2016). “ Rethinking the Inception architecture for computer vision”, in: Proceedings of the IEEE/CVF Conference on Computer Vision and Pattern Recognition (Las Vegas, NV: IEEE), 2818–2826.

[B24] Tan M. Le Q. V. (2019). “ EfficientNet: Rethinking model scaling for convolutional neural networks”, in: Proceedings of the 36th International Conference on Machine Learning (Long Beach, California: PMLR), 6105–6114.

[B25] Watkins C. J. C. H. Dayan P. (1992). Q-learning. Mach. Learn. 8, 279–292. doi: 10.1007/BF00992698 30311153

[B26] Williams R. J. (1992). Simple statistical gradient-following algorithms for connectionist reinforcement learning. Mach. Learning. 8, 229–256. doi: 10.1023/a:1022672621406 41886696

[B27] Xu S.-H. Wang S. (2025). An intelligent identification for pest and disease detection in wheat leaf based on environmental data using multimodal data fusion. Front. Plant Sci. 16, 1608515. doi: 10.3389/fpls.2025.1608515 40933711 PMC12417405

[B33] Zang H. Wang Y. Wang S. Ren S. Yang Y. Zhang J. . (2026). Study on automatic detection of wheat spike grain number based on deep learning. Front. Plant Sci. 17, 1724501. doi: 10.3389/fpls.2026.1724501 41821941 PMC12975923

